# Integrating serum pharmacology, network pharmacology, and molecular biology analysis to reveal the mechanisms of Baihe Dihuang decoction in treating Alzheimer’s disease

**DOI:** 10.3389/fmicb.2026.1765044

**Published:** 2026-07-02

**Authors:** Xicai Liang, Tianyi Ning, Ying Wang, Libin Zhan

**Affiliations:** 1 College of Laboratory Animal Medicine, Liaoning University of Traditional Chinese Medicine, Shenyang, China; 2 Key Laboratory of Ministry of Education for TCM Viscera-State Theory and Applications, Liaoning University of Traditional Chinese Medicine, Shenyang, China; 3 National and Local Joint Engineering Laboratory for Integrated Chinese and Western Medicine Prevention and Treatment Technology on Cardio-Brain Diseases, Liaoning University of Traditional Chinese Medicine, Shenyang, China

**Keywords:** 16S rRNA sequencing, Baihe Dihuang decoction, gut-brain axis, network pharmacology, serum pharmacochemistry

## Abstract

**Aim:**

This study aimed to evaluate the potential neuroprotecive effects of Baihe Dihuang Decoction (BDD) in APP/PS1 double-transgenic (TG) mice and to investigate the role of the gut-brain axis (GBA) using an integrated approach combining serum pharmacology, network pharmacology, and molecular biology.

**Materials and methods:**

The blood-borne bioactive components of BDD were initially identified using UPLC-Q-Orbitrap HRMS. Subsequently, network pharmacology was employed to prioritize key therapeutic targets and elucidate the primary pathways underlying the anti-Alzheimer’s disease (AD) effects of BDD. The neuroprotective efficacy of BDD in TG mice was systematically evaluated using the morris water maze (MWM) test, histopathological observation (HE staining), transmission electron microscope (TEM) test, and ELISA-based inflammatory cytokine assays. The potential mechanisms were further elucidated by integrating network pharmacology with 16S ribosomal RNA (16S rRNA) sequencing. Finally, molecular docking and Western blotting (WB) were performed to validate the interactions within the identified pathways.

**Results:**

A total of 49 BDD-derived compounds were identified in serum samples. Network pharmacology revealed 116 common targets of BDD against AD. Remarkably, KEGG analysis highlighted 57 signaling pathways potentially involved in the anti-AD effects of BDD. Pharmacodynamic analysis showed that BDD ameliorated cognitive impairment in TG mice, mitigated pathological damage, and suppressed the release of IL-6, IL-1β, and TNF-α in the colon, brain, and serum. Moreover, 16S rRNA sequencing indicated that BDD modulated gut microbiota (GM) structure and restored intestinal flora imbalance in TG mice. Integrative analysis of network pharmacology and GM analysis identified several Key pathways (FoxO, MAPK, PI3K-Akt, HIF-1, Th17, IL-17, and Toll/Imd) and core anti-AD targets (TLR4, PTGS2, SIRT1, BDNF, NF-κB, STAT3, JAK2, EGFR, GSK3β, and CD86). Molecular docking results showed that the five complexes with the lowest docking scores (TLR4-salidroside, NF-κB-glabranin, TRKB-alantolactone, PTGS2-3′_4′_dihydroxyflavone, and SIRTI-abietic acid) exhibited strong binding affinity. QSAR and WB analyses further demonstrated the modulatory effects of BDD on these five core targets.

**Conclusion:**

This study demonstrated that BDD effectively restored GM structure and ameliorated cognitive impairment in TG mice, thereby exerting therapeutic effects against AD. These findings support BDD as a potential traditional Chinese medicine (TCM) strategy for AD treatment.

## 1 Introduction

Alzheimer’s disease (AD) is a common neurodegenerative disease (ND) of the central nervous system (CNS) that primarily affects individuals over 65 years of age. Due to its protracted course and multifactorial pathogenesis, no truly effective therapy has yet been developed. Patients experience progressive decline in memory and mobility, along with the gradual loss of acquired knowledge-burdens that reverberate through families and society at large (Duggan et al., 2025). Traditionally considered confined to the brain, AD was once viewed as a strictly cerebral condition, disconnected from the rest of the body. However, the prevailing perspective is shifting toward a systemic, whole-organism paradigm. Mounting evidence now firmly links the onset of AD to an imbalance in the gut microbiota (GM) (Zhang et al., 2023; Varesi et al., 2022). The bidirectional modulation of the gut-brain axis (GBA) underscores that GM homeostasis plays a pivotal role in the initiation and progression of the characteristic cognitive decline and behavioral disturbances in AD. Restoring microbial eubiosis can dampen aberrant metabolite surges, quells peripheral and neuroinflammation, and ultimately slow neurodegeneration (Loh et al., 2024; Doifode et al., 2021; Megur et al., 2020). Therefore, elucidating the mechanistic role of the microbiota in AD promises will not only provide novel therapeutic and preventive strategies but also enable earlier and more precise diagnosis.

The recently proposed concept of GBA indicates bidirectional regulation between the GM and brain CNS. GM can significantly affect brain function and behavior through three primary pathways (immune, neuroendocrine, and vagus nerve pathways). Central to AD pathogenesis is immune activation: once pathology or systemic inflammation compromises the blood–brain barrier (BBB), circulating cytokines flood the brain and fuel a self-perpetuating neuroinflammatory cascade (Kim et al., 2021; Zou et al., 2023). Importantly, the metabolites produced by GM imbalance [e.g., lipopolysaccharides (LPS)] can disrupt tight junctions of enterocytes and increase intestinal permeability, thereby inducing inflammatory reactions in the bloodstream. This subsequently damages the BBB and exposes nerve cells to GM-released metabolites, ultimately leading to neuroinflammation, neuronal damage, and degeneration (Marizzoni et al., 2023).

The GM and immune function are reciprocally regulated during the onset and progression of AD. Dysbiosis increases intestinal permeability, ignites systemic immune activation, and drives a marked upregulation of inflammatory markers (Yang et al., 2020; Wei et al., 2023). Additionally, the GM can produce various neuroactive molecules [e.g., dopamine (DA) and 5-hydoxytryptamine], which contribute to GM dysbiosis and aggregation, thereby damaging the BBB, promoting neuroinflammation and neurodegeneration, and ultimately facilitating AD onset (Loh et al., 2024; Ohara and Hsiao, 2025). Nevertheless, the precise mechanisms by which GM dysbiosis drives AD pathogenesis remain largely elusive. Clarifying how the BGA orchestrates this process will establish a mechanistic foundation for novel preventive and therapeutic strategies against AD.

Traditional Chinese medicine (TCM) is characterized by its multi-constituent and multi-target mechanisms, which offers extensive therapeutic advantages. Growing evidence suggests that herbal medicines can restore GM balance, indicating their potential value in managing NDs such as AD (Ma et al., 2024; Kim et al., 2023). In particular, TCM formulas with heat-clearing properties have shown considerable promise in AD treatment (Hou et al., 2024; Wang et al., 2022). Baihe Dihuang Decoction (BDD), a well-known TCM formula, is primarily used to treat neurological disorders (Ran et al., 2023; Tang et al., 2024). Studies have demonstrated that BDD effectively suppresses inflammatory cascade in depressed mice (Mao et al., 2024), Nonetheless, there is currently no research focusing on the efficacy of BDD against AD. BDD consists of two herbs: Baihe (*Lilii Bulbus*) and Dihuang (*Rehmanniae Radix*), both of which have been reported to possess potential anti-AD effects. For example, it has been shown that Baihe saponins effectively inhibits the increase in nitric oxide (NO), prostaglandinE2 (PGE2), and inflammatory factors in the brains of LPS-induced mice (Park et al., 2023). Moreover, Baihe polysaccharides can regulate GM structure and reduce the release of inflammatory factors in LPS-induced mice (Lim et al., 2020). Additionally, *verbascoside*, an active ingredient in Dihuang, can reshape GM composition and attenuate pathological brain damage, thereby alleviating cognitive dysfunction of db/db mice (Ran et al., 2023; Su et al., 2023).

In summary, this study investigated the cognitive benefits of BDD in APP/PS1 double-transgenic (TG) mice and elucidates the pivotal mechanisms centered on GM modulation and neuroinflammation suppression. These findings are expected to reveal how BDD leverages microbiota remodeling to exert therapeutic effects against AD.

## 2 Methods and materials

**Figure 1:**
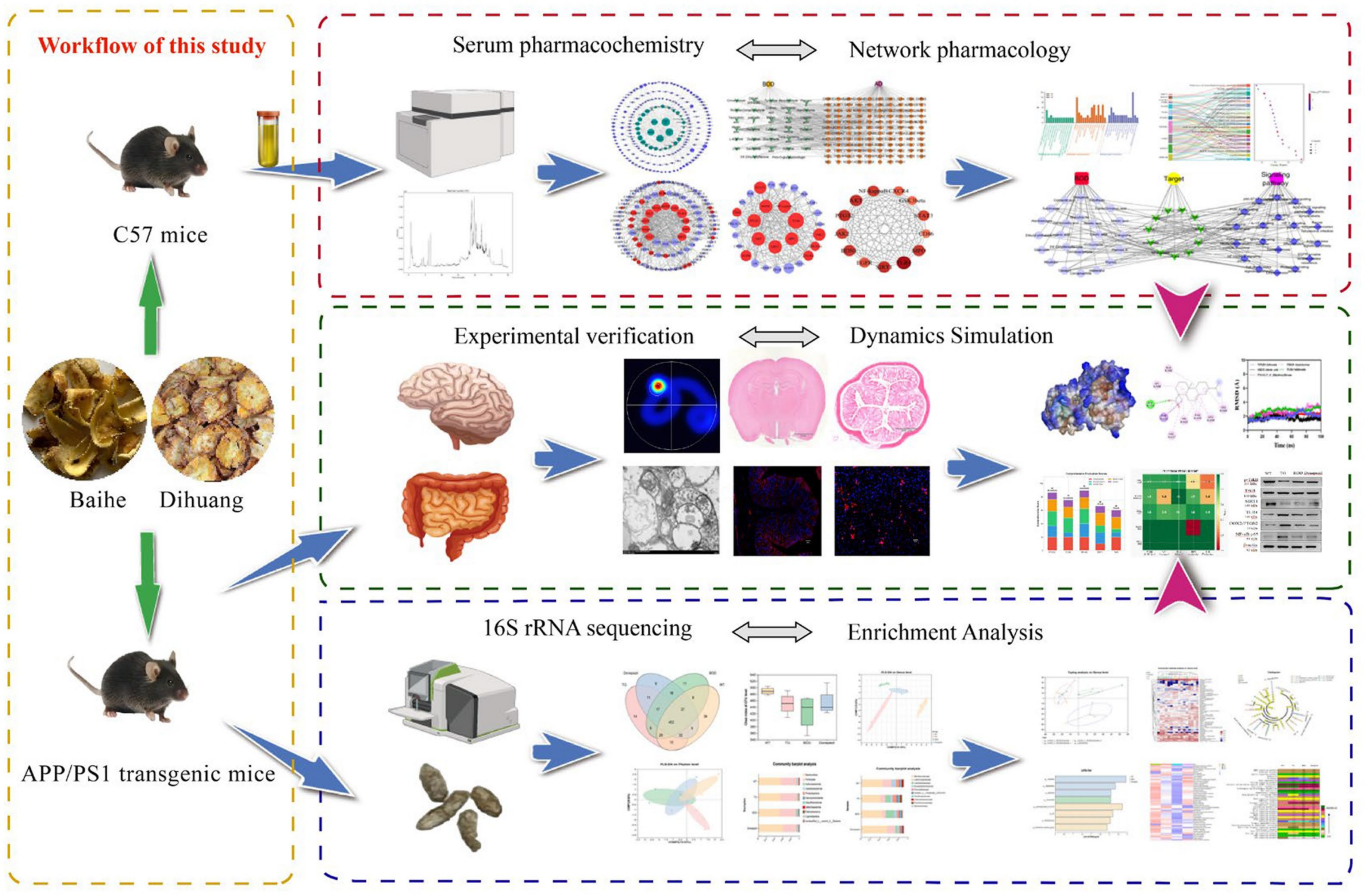
Workflow of the present study.

The workflow is depicted in Figure 1.

### 2.1 Materials, reagents and animals

#### 2.1.1 Materials and reagents

Acetonitrile, formic acid, methanol (chromatography grade) were supplied by Merck (Darmstadt, Germany). Baihe (*Lilium brownii* var. *viridulum Baker*) and Dihuang (*Rehmanniae radix*) were obtained from the Liaoning University of TCM. Donepezil hydrochloride (Anlishen, 5 mg, batch No. 2202013) was manufactured by Eisai (China) Pharmaceutical Co., Ltd. Enzyme-linked immunosorbent assay (ELISA) kits for interleukin (IL)-6, IL-1β, and tumor necrosis factor-α (TNF-α) (lot Nos. 203049M, 203063M, 202412M) were obtained from Ruixin Biology. Mouse acetylcholinesterase (AChE), glutathione (GSH), and DA kits (lot Nos. A024-1-1, A006-2-1, H170-1-1) were supplied by Nanjing Jiancheng Bioengineering Institute. The anti-ZO1 tight junction protein Rabbit (ZO-1) antibody (GB115686), Occludin Rabbit (GB111401), GFAP Rabbit antibody (GB15100-50) and Iba1Rabbit antibody (GB113502-50) were purchased from Servicebio, anti-cyclooxygenase-2 (COX2)/prostaglandin-Endoperoxide Synthase 2 (PTGS2) (A25901) antibody and anti-sirtuin 1 (SIRT1) antibody (A27256) were supplied by ABclonal Technology Co., Ltd., The anti-toll-like receptor 4 (TLR4) antibody (19811-1-AP) was supplied by the Proteintech Group, Inc.; anti-p-tropomyosin receptor kinase B (TrkB) antibody (bsm-52213R), TrkB antibody (bs-0175R) and anti-nuclear factor kappa-B (NF-κB) p65 (bs-20160R) antibody were purchased from Bioss Biotechnology Co., Ltd., anti-cyclooxygenase-2 (COX2)/prostaglandin-Endoperoxide Synthase 2 (PTGS2) (A25901) antibody and anti-sirtuin 1 (SIRT1) antibody (A27256) were supplied by ABclonal Technology Co., Ltd.

#### 2.1.2 Animals

This study was approved by the Institutional Animal Care and Use Committee of Liaoning University of TCM (21000042023077). TG mice and wild-type (WT) mice (50 TG mice and 16 WT mice, 6 months old, half male and half female, weighed 22 ± 5 g) were obtained from Beijing Sipeifu Biotechnology Co., Ltd. [animal license No. SYSK (Liao) 2019-0004]. All animals were housed in a specific pathogen-free barrier system with free access to drinking water and food.

### 2.2 Serum phamacochemistry

#### 2.2.1 Preparation of serum sample

WT mice were randomly grouped to the control and BDD-treated groups (*n* = 3 per group). Mice in the BDD group received 40 g/kg BDD solution by gavage for 7 days, while control mice received an equal volume of purified water. Whole-blood samples were collected from the retro-orbital plexus at 1 and 1.5 h after the final gavage. Serum was obtained by allowing the blood samples to coagulate at room temperature (RT) for 30 min, followed by centrifugation (3,000 rpm, 10 min). The serum samples were subjected to compositional characterization using ultra-high-performance liquid chromatography-high resolution mass spectrometry (UPLC-Q-Orbitrap HRMS) (Qiu et al., 2025).

#### 2.2.2 UPLC-Q-Orbitrap HRMS analysis

A Triple TOF 5600 system interfaced with a Waters H-Class UPLC was utilized for analysis (AB Sciex, Waters, United States). Separation was performed on a Waters acquity UPLC HSS T3 column at 40 °C with a flow rate of 0.25 mL/min. The mobile phase consisted of eluent A (0.1% formic acid in aqueous solution) and eluent B (0.1% fomic acid in acetonitrile). Gradient elution was performed as follows: initial hold at 5% B for 1.5 min; linear rise from 10 to 40% B over 2.5–14 min; further increase to 95% B from 14 to 24 min; hold at 95% B for 3 min; and re-equilibration at 5% B from 27.1 to 30 min.

Mass spectrometry was conducted in fast polarity-switching mode, alternating between positive and negative ionization, with data acquisition over an m/z range of 50–120. The experimental parameters were as follows: nebulizer gas pressure, 60 psi; gas temperature, 550 °C; ion spray voltage, 5,500 V (−4,500 V); and collision energy, 45 V, Data acquisition was performed utilizing Analyst TF 1.7 software (AB Sciex).

### 2.3 Network-pharmacology analysis

#### 2.3.1 Collection of potential targets for BDD and screening of AD-related genes

Serum-derived active constituents of BDD were further screened against the PubMed^1^ and Traditional Chinese medicine systems pharmacology database and analysis platform (TCMSP^2^). AD-associated genes were retrieved from the comparative toxicogenomics database (CTD^3^). The overlapping targets between BDD constituents and AD were mapped using Cytoscape (version 3.9.1).

#### 2.3.2 Mapping the “ingredient-target-disease” network

Core constituents and targets were identified by topological analysis using the Network Analyzer plugin in Cytoscape. This identification was based on three critical network metrics: degree, closeness centrality, and betweenness centrality, evaluated within the visualized “ingredient-target-disease” network.

#### 2.3.3 Core targets identification and enrichment analysis

Protein–protein interaction (PPI) data were retrieved from the STRING database.^4^ To ensure reliability, only interactions with a confidence score above 0.7 were selected for subsequent network construction in Cytoscape. The Analyze Network plugin was used to quantitatively characterize the network topology. The core targets were submitted to the Metascape platform for Gene Ontology (GO) and Kyoto Encyclopedia of Genes and Genomes (KEGG) enrichment analyses. The Benjamini-Hochberg method was applied for false discovery rate correction, with a threshold of *p* < 0.05.

### 2.4 Experimental verification

#### 2.4.1 Treatment and group allocation

BDD consists of Baihe (240 g) and fresh Dihuang (240 g). All herbal pieces were ground into powder, soaked in 10 volumes of water for 0.5 h, and then decocted for 1 h. The first decoction was filtered out, and another 10 volumes of water were added for a second decoction (1 h). The two batches of herbal decoction were mixed and concentrated. The final concentrations were prepared as 0.5 g/mL (low), 1.0 g/mL (medium), and 2.0 g/mL (high), and stored at 4 °C for future use. TG mice were randomly allocated into 3 groups (5 males and 5 females per group, n = 10): TG group, BDD-treated group (low, medium, and high doses of 10, 20, and 40 g/kg, respectively), and donepezil group (0.65 mg/kg). The low-, medium-, and high-dose BDD groups received a daily gavage of 0.5 mL of the corresponding BDD formulation for 4 weeks, while the WT group received the same volume of purified water. The morris water maze (MWM) test was conducted during the third week of intragastric treatment (Xiao et al., 2022).

#### 2.4.2 MWM test

The MWM test was performed using a circular tank (120 cm In diameter) filled with opaque water (25 °C), with distal visual cues placed around the tank. Mice were trained to locate a colored platform (15 cm × 15 cm) from different quadrants in the pool. The experiment lasted 6 days, including a 5-day training phase with twice-daily sessions (10 training trials in total). To assess spatial memory, escape latency (the time taken by each mouse to locate and climb onto the platform) was first recorded. In the subsequent spatial probe test, the platform was removed, and the mice were reintroduced into the maze. Memory retention was quantified by tracking two parameters: the frequency of platform crossings and duration in the target quadrant (Xiao et al., 2022).

#### 2.4.3 HE staining

Tissue sections (proximal colon and brain) were equilibrated at RT for 10 min, cleared in xylene, sequentially immersed in graded ethanol solutions (100, 95, 90, 80%), and finally rinsed twice with distilled water. After hematoxylin staining for 1 min, the sections were briefly washed in distilled water, differentiated in 1% HCl-ethanol for 10–20 s, and rinsed once more. Afterwards, the sections were blued in 0.5% ammonia water for 10 s, counterstained with eosin for 3–5 s, then dehydrated through ascending ethanol series (90, 95, 100%) and cleared in xylene for 5 s. Finally, the sections were mounted with neutral resin. Tissue morphology was examined under a light microscope. Histological scoring was conducted according to criteria established in previous studies (Tao et al., 2024), focusing on three independent parameters: (a) severity of inflammation (score 0–3: none, mild, moderate, severe), (b) degree of damage (score 0–3: none, mucosa, mucosa and submucosa, transmural), and (c) colon crypt damage (score 0–4: none, basal one-third damage, basal two-thirds damage, surface epithelium intact, entire crypt and epithelial loss). Brain histomorphology was scored as follows (score 0–4): 0, normal morphology; 1, loosely arranged cells; 2, cell swelling or nuclear morphological changes; 3, cell necrosis with partial disruption of tissue architecture; 4, extensive cell necrosis with complete loss of normal structure. Each parameter score was multiplied by a factor reflecting the percentage of tissue involvement (x 1, 0–25%; x 2, 26–50%; x 3, 51–75%; and x 4, 76–100%), and the total score was obtained by summing these values.

#### 2.4.4 Transmission electron microscope imaging

Upon completion of BDD treatment, randomly selected samples from hippocampus (CA1) were collected for ultrastructural examination by transmission electron microscope (TEM) imaging (Kim et al., 2024). Specifically, after deep anesthesia with 10% sodium pentobarbital, the hippocampal tissue was rapidly excised, diced into 1 mm^3^ blocks, and fixed in 1% OsO₄ at RT for 2 h. Following dehydration and resin embedding, 70 nm ultrathin sections were cut, sequentially contrast-stained with 2% uranyl acetate and lead citrate (15 min), and imaged on a Hitachi HT7700 TEM (Tokyo, Japan).

#### 2.4.5 Enzyme linked immunosorbent assay

The proximal colon tissue fragments (100 mg) were homogenized in ice-cold 0.9% saline. After centrifugation (12, 000 rpm, 10 min), the cleared lysates were assayed for IL-6, IL-1β, and TNF-α using commercial ELISA kits. Blood samples were centrifuged (3, 000 rpm, 10 min), and the resulting serum was analyzed for IL-6, IL-1β, TNF-α, AChE, GSH, and DA levels using the same kit format.

#### 2.4.6 Immunofluorescence staining

The colon and brain tissue sections were deparaffinized and subsequently fixed with 4% PFA for 30 min. Following two washes with PBS, the sections were permeabilized using 0.5% Triton X-100 at room temperature for 5 min. The sections were then incubated with primary antibodies overnight at 4 °C. The primary antibodies used were as follows: ZO-1 (1:1000), Occludin (1:800), GFAP (1:1000), Iba1 (1:1000). Subsequent to washing twice with PBS, the samples were incubated with Cy3-conjugated secondary antibodies for 1.5 h at room temperature in the dark. Nuclei were counterstained with DAPI for 5 min following two final PBS washes. An anti-fade mounting medium was applied, and images were captured using a fluorescence microscope.

### 2.5 16S ribosomal RNA sequencing analysis

#### 2.5.1 Fecal sample collection

After a 12-h fasting period following the final administration, feces were collected by gentle abdominal compression near the tail root and the end of rectum. The extracted feces were collected into sterile centrifuge tubes, and paired-end sequencing was performed using the high-throughput Illumina Miseq PE300 platform.

#### 2.5.2 GM analysis

The 16S ribosomal RNA sequencing (16S rRNA) gene was sequenced, and operational taxonomic units (OTUs) were picked to profile α- and β-diversity and to compare community composition across groups. Using Vsearch (v2.7.1), high-quality reads were grouped into OTUs at a 97% sequence identity cutoff. The RDP classifier algorithm, utilizing the silva 128 database with a 70% confidence threshold, provided species classification information for each OTU. α-diversity index analysis was performed using QIIME1 (v1.8.0) software, while β-diversity analysis and Venn diagram generation were conducted using *R* (Qian et al., 2023).

### 2.6 Molecular docking verification

SDF files of the primary compounds In BDD were downloaded from the TCMSP database, while AD-related targets were extracted from the PDB database.^5^ The 3D structures in SDF format were converted into Mol2 format using OpenBabelGui software. Subsequently, the large proteins underwent hydrogenation, dehydration, and other necessary pretreatments in AutoDockTools. The main ingredients and hub genes were then docked utilizing Autodock Vina to determine binding energies. The molecular docking results were visualized and PNG images were generated using Discovery Studio™.

### 2.7 Molecular dynamics simulations

Molecular dynamics simulations were performed using GROMACS 2022. Receptor topologies were built with pdb2gmx plus AutoFF, assigning the AMBER14SB to the protein and GAFF2 to the ligands. Each complex was immersed in a 1-nm TIP3P water cube and neutralized using genion. Long-range electrostatics were handled by the particle mesh Ewald (PME) method with a 1-nm cutoff. Bond lengths were constrained using the SHAKE algorithm, and a leap-frog integrator advanced the trajectory with a 1-fs time step. After energy minimization consisting of 3, 000 steepest-descent and 2, 000 conjugate-gradient, production simulations were launched.

### 2.8 Quantitative structure–activity relationship analysis

To further validate the interaction potential between the active ingredients of BDD and their targets at the molecular level, this study employed quantitative structure–activity relationship (QSAR) methods to systematically evaluate the five active compounds mentioned above. The training datasets were retrieved from the ChEMBL database, collecting compounds with explicit bioactivity data (IC50 values) for each target: 447 compounds for PTGS2, 383 for TLR4, 454 for NF-κB-related targets, 386 for SIRT1, and 330 for TrkB. All IC50 values were converted to pIC50 values for subsequent modeling analysis. Molecular descriptors were calculated using the RDKit cheminformatics toolkit, including physicochemical parameters such as molecular weight, octanol–water partition coefficient (logP), topological polar surface area (TPSA), number of hydrogen bond donors, number of hydrogen bond acceptors, and number of rotatable bonds. Molecular fingerprints were generated using the Morgan circular fingerprint algorithm (radius = 2, bits = 2048) to encode structural information. QSAR model construction adopted an ensemble learning strategy that combined gradient boosting decision tree and random forest algorithms. A pIC50 ≥ 5.5 was used as the activity threshold to classify active and inactive compounds. The dataset was randomly partitioned into training and test sets at an 8:2 ratio, and five-fold cross-validation was employed to assess generalization capability, with the area under the receiver operating characteristic (ROC) curve (AUC) serving as the primary evaluation metric. Drug-likeness of the compounds was assessed based on Lipinski’s Rule of Five and Veber’s rules.

### 2.9 Western blotting

Brain tissues were homogenized using RIPA lysis buffer containing protease and phosphatase inhibitors. The tissue samples were placed on ice for 30 min and then centrifuged (4 °C, 15 min, 12,000 rpm). The supernatant was collected, and protein concentrations were determined using a BCA protein assay kit. Equal amounts of protein were separated by SDS-PAGE and then transferred to membranes. After blocking in TBST with either 5% skimmed milk or 5% BSA, the membranes were incubated (overnight, 4 °C) with primary antibodies on a shaker. The primary antibodies used were as follows: TLR4 (1:800), p-TrkB (1:2000), NF-κB p65 (1:2000), COX2/PTGS2 (1:1000), and SIRT1 (1:1000). Subsequently, the membranes were incubated (2 h, 4 °C) with the secondary antibody with shaking. Protein bands were visualized using ECL. ImageJ software was employed to quantify the gray values.

### 2.10 Statistical analysis

Data were expressed as mean ± standard error (SEM) and analyzed using GraphPad Prism software (version 8.0). Before comparative analyses, the normality of data distribution was assessed using the Shapiro–Wilk test. For data with normal distribution, intergroup and intragroup comparisons were evaluated by analysis of variance followed by Tukey’s *post-hoc* test. Correlations between two variables were examined using Pearson correlation analysis. A *p* value of < 0.05 was indicative of a statistically significant difference.

## 3 Results

### 3.1 Identification of BDD components in serum

**Figure 2:**
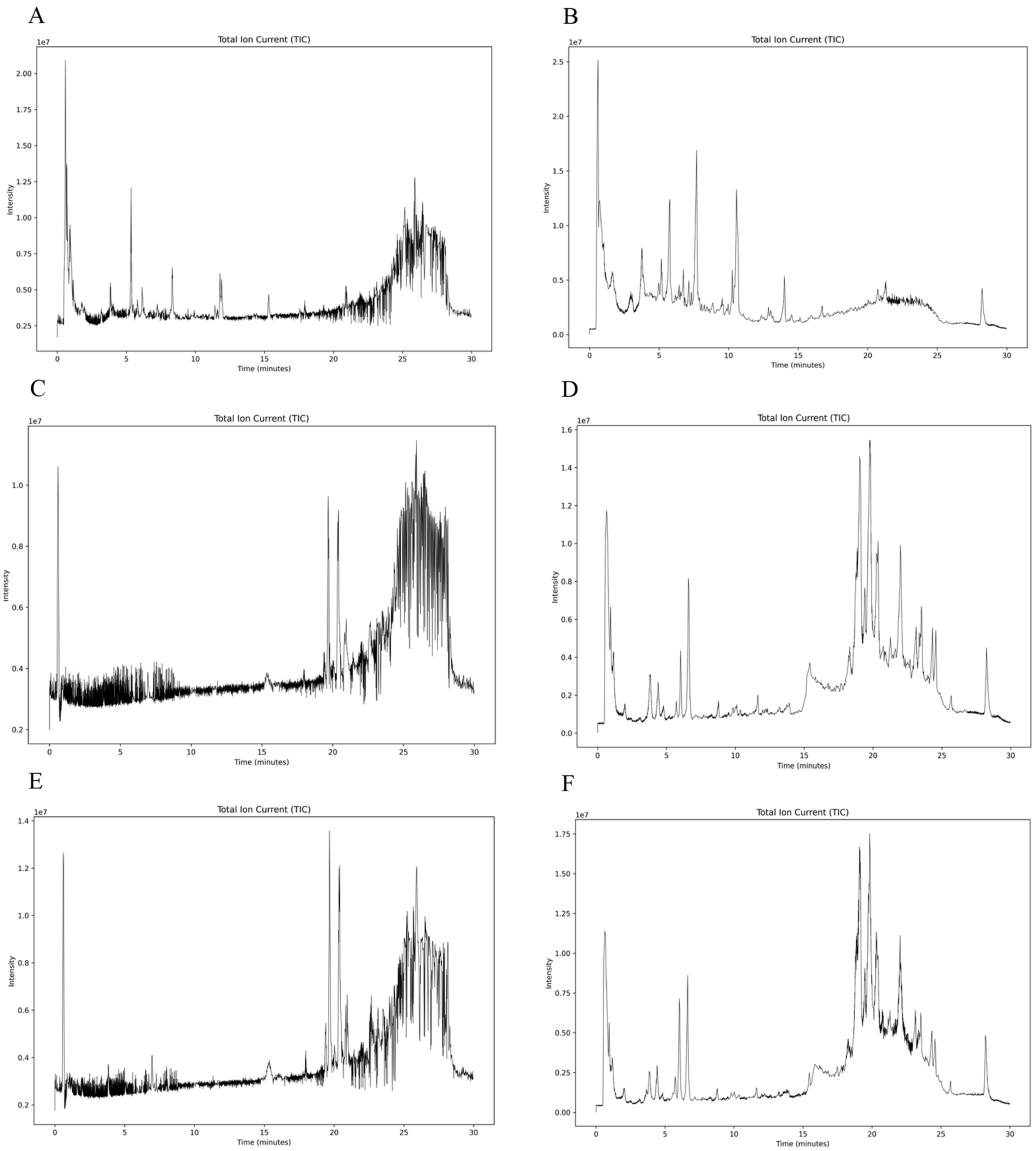
TICs of positive and negative ion modes. **(A,B)** BDD solution; **(C,D)** blank serum; **(E,F)** post-BDD administration serum.

Using mzCloud, ChemSpider, and relevant literature data (Hardinsyah et al., 2023), a total of 49 blood-absorbed BDD constituents were identified, including 19 phenylpropanoids, 16 lipids, 2 organic acids, 2 alkaloids, and 10 other compounds (Table 1). These compounds were used for subsequent network pharmacology analysis. Figure 2 displays representative total-ion chromatograms (TICs) of blank serum, BDD-containing serum, and post-BDD administration serum.

**Table 1:** Characterization of BDD-derived blood-absorbed components.

NO.	Peak ID	Title	RT (min)	theoretical m/z	Precursor m/z	Adduct	Formula
1	1	Dibutyl phthalate	0.4230333	279.159086	279.1557	[M+H]+	C16H22O4
2	17	Histamine	0.5259833	112.086923	112.0854	[M+H]+	C5H9N3
3	551	Justicidin B	0.6510667	365.101966	365.1031	[M+H]+	C21H16O6
4	670	Uracil	0.9840167	113.034554	113.0335	[M+H]+	C4H4N2O2
6	764	Cinnamaldehyde	1.6132	133.064791	133.0631	[M+H]+	C9H8O
7	768	Cinnamamide	1.6132	148.07569	148.0766	[M+H]+	C9H9NO
8	836	Musk ketone	4.599167	295.128849	295.1288	[M+H]+	C14H18N2O5
9	838	Hippuric acid	4.958467	180.06552	180.063	[M+H]+	C9H9NO3
10	852	Senecionine	5.7492	194.08117	194.0807	[M+H]+	C10H11NO3
11	1,191	Phthalic anhydride	15.83657	149.023321	149.0234	[M+H]+	C8H4O3
12	1,237	Alantolactone	16.42553	233.153606	233.1531	[M+H]+	C15H20O2
13	1,291	Pterosin G	17.76815	235.132871	235.13	[M+H]+	C14H18O3
14	1,293	Thymol	17.7734	151.111741	151.1095	[M+H]+	C10H14O
15	1,338	Carpachromene	18.53235	337.107051	337.1059	[M+H]+	C20H16O5
16	1,686	Curcumenol	19.93435	235.169256	235.1686	[M+H]+	C15H22O2
17	1,952	Glabranin	20.50467	325.143436	325.1447	[M+H]+	C20H20O4
18	1,957	Pterosin B	20.5246	219.137956	219.136	[M+H]+	C14H18O2
19	2,042	Lauric acid	20.92572	201.184906	201.1834	[M+H]+	C12H24O2
20	2,262	Abietic acid	22.48698	303.231856	303.2303	[M+H]+	C20H30O2
21	2,327	Diosgenin	22.92482	415.320671	415.3213	[M+H]+	C27H42O3
22	2,334	Swainsonine	22.97302	174.11247	174.1105	[M+H]+	C8H15NO3
23	2,337	Psilotin	22.97302	353.123096	353.1207	[M+H]+	C17H20O8
24	2,385	Cinanserin	23.11708	341.16821	341.1724	[M+H]+	C20H24N2OS
25	2,483	Emetine Dihydrochloride	23.54217	553.25944	553.2534	[M+H]+	C29H42Cl2N2O4
26	2,682	11,12-Methylenedioxykopsinaline	24.49928	399.191449	399.1916	[M+H]+	C22H26N2O5
27	2,817	Taurocholic acid	25.12252	516.298951	516.2959	[M+H]+	C26H45NO7S
28	3,430	Coniferylaldehyde	28.12262	179.070271	179.0684	[M+H]+	C10H10O3
29	3,476	Tyramine	28.14292	138.09134	138.0917	[M+H]+	C8H11NO
30	146	L-Arginine	0.6006167	173.1044	173.1036	[M−H]−	C6H14N4O2
31	350	Crotonic acid	0.6868333	85.029504	85.02932	[M−H]−	C4H6O2
32	455	Maleic acid	0.7297834	115.003684	115.004	[M−H]−	C4H4O4
33	826	Catalpol	1.048533	361.114024	361.1146	[M−H]−	C15H22O10
34	865	Adenine	1.083583	134.047219	134.0462	[M−H]−	C5H5N5
35	1,064	Shanzhiside	1.507067	391.124589	391.126	[M−H]−	C16H24O11
36	1,126	Trans-Cinnamic acid	1.980867	147.045154	147.0445	[M−H]−	C9H8O2
37	1,184	Geniposidic acid	3.049417	373.114024	373.1133	[M−H]−	C16H22O10
38	1,209	Loganic acid	3.600683	375.129674	375.1299	[M−H]−	C16H24O10
39	1,234	Homovanillic acid	3.791767	181.050634	181.051	[M−H]−	C9H10O4
40	1,377	Riboflavin	3.903767	375.13101	375.1284	[M−H]−	C17H20N4O6
41	1,646	Sinapoylhexoside	5.437284	385.114024	385.1133	[M−H]−	C17H22O10
42	2,062	3′,4’-Dihydroxyflavone	7.7178	253.050634	253.0491	[M−H]−	C15H10O4
43	2,087	Rhoifolin	8.069883	577.156284	577.1572	[M−H]−	C27H30O14
44	2,314	Tauro	10.6301	498.289484	498.2897	[M−H]−	C26H45NO6S
45	3,437	12a-HYDROXY-5-DEOXYDEHYDROMUNDUSERONE	18.63343	341.103064	341.1035	[M−H]−	C19H18O6
46	4,406	Abietic acid	20.21532	301.217304	301.2183	[M−H]−	C20H30O2
47	4,708	Corosolic acid	21.15235	471.347984	471.3482	[M−H]−	C30H48O4
48	5,153	prim-O-Glucosylcimifugin	23.13512	467.155889	467.151	[M−H]−	C22H28O11
49	5,219	Rosarin	23.43218	427.160974	427.163	[M−H]−	C20H28O10

### 3.2 Network pharmacology analysis

#### 3.2.1 Prediction of anti-AD targets for BDD serum component

**Figure 3:**
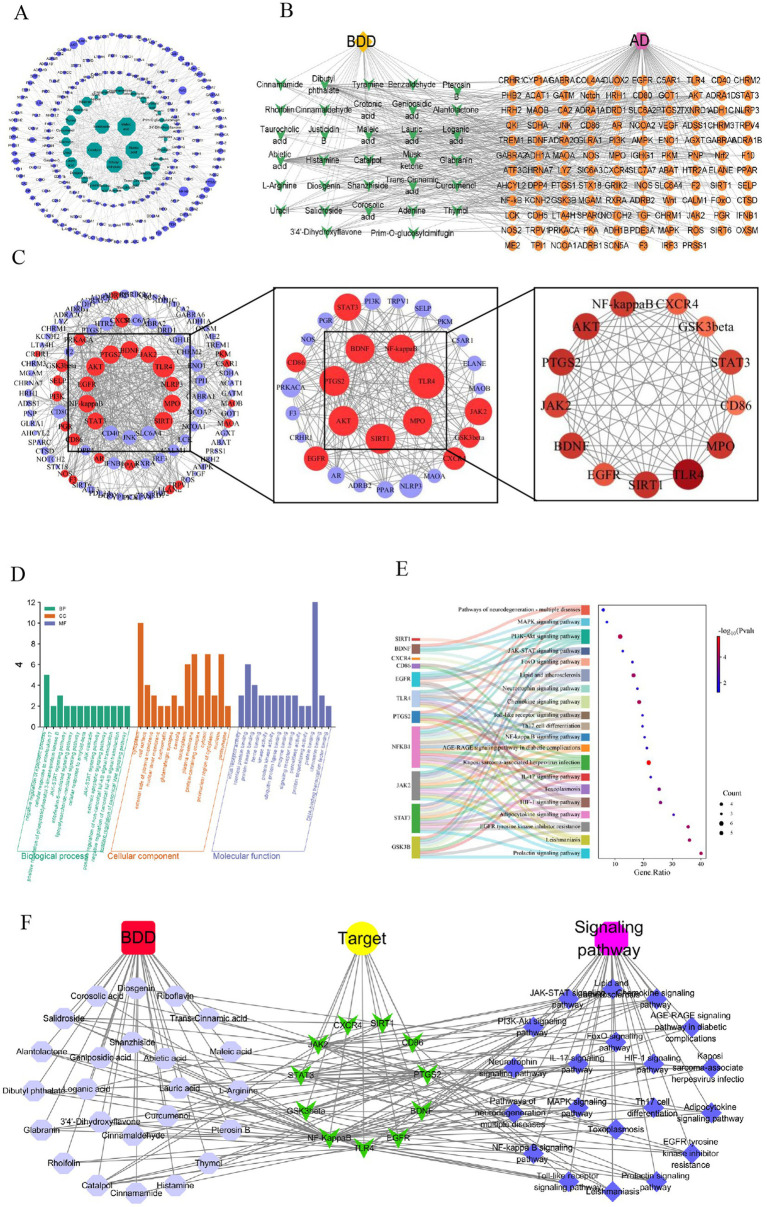
The network pharmacology analysis results. **(A)** Identification of the potential targets of BDD through constructing a PPI network; **(B)** the compound-AD targets PPI network; **(C)** screening of the core targets; **(D)** GO enrichment analysis of BDD anti-AD targets; **(E)** KEGG enrichment analysis of BDD anti-AD targets; **(F)** the components-targets-pathways network of BDD against AD.

Comprehensive mining of the TCMSP and PubMed databases yielded 434 targets linked to the 49 blood-borne constituents with putative anti-AD. After deduplication, the CTD repository contributed 29,056 AD-associated genes. After intersecting these two datasets, 118 core targets were identified, which may underpin the anti-AD efficacy of the 49 active components of BDD (Figures 3A,B).

#### 3.2.2 Construction of the PPI network

The 118 overlapping targets were imported into the STRING database. The obtained interaction data were used to construct and visualize a PPI network using Cytoscape. As shown in Figure 3C, this network comprised 106 nodes (representing target genes) and 662 edges (corresponding to protein interactions). The degree value of a node indicated the strength and proximity of its interactions with other proteins, with higher degrees suggesting more significant interactions. Based on network topology analysis, 13 node proteins with degrees exceeding the median threshold of 13.5 were identified. These proteins include TLR4, PTGS2, AKT, SIRT1, BDNF, MPO, NF-κB, STAT3, JAK2, EGFR, CXCR4, GSK3β, and CD86. These targets converge on inflammatory signaling, immune modulation, cell-proliferation control, apoptosis regulation, and metabolic homeostasis—processes that collectively drive AD pathophysiology.

#### 3.2.3 Enrichment analysis

Subsequently, 13 common targets were enriched and examined to understand how BDD exerted its effects in AD. The Metascape database was used to perform GO analysis on these crucial common targets. The results revealed 93 GO terms associated with biological process (BP), 14 with cellular component (CC), and 20 with molecular function (MF). A bar plot (Figure 3D) was generated to display the 14 most significantly enriched terms (*p* < 0.01) from each of the above three categories.

KEGG analysis of the BDD-AD target network identified 57 significantly enriched pathways (*p* < 0.05). Among the top 20 pathways were AMPK, FoxO, PI3K-Akt, HIF-1, IL-17, MAPK, and cAMP signaling (Figure 3E). A total of 11 targets were identified as central to these top 20 pathways, suggesting their importance for the anti-AD efficacy of BDD. A component-target-pathway network was constructed using Cytoscape to visualize the relationships between the active components in BDD and their roles within this network (Figure 3F).

### 3.3 Pharmacodynamic study of BDD

#### 3.3.1 Effects of BDD on cognitive deficits in TG mice

**Figure 4:**
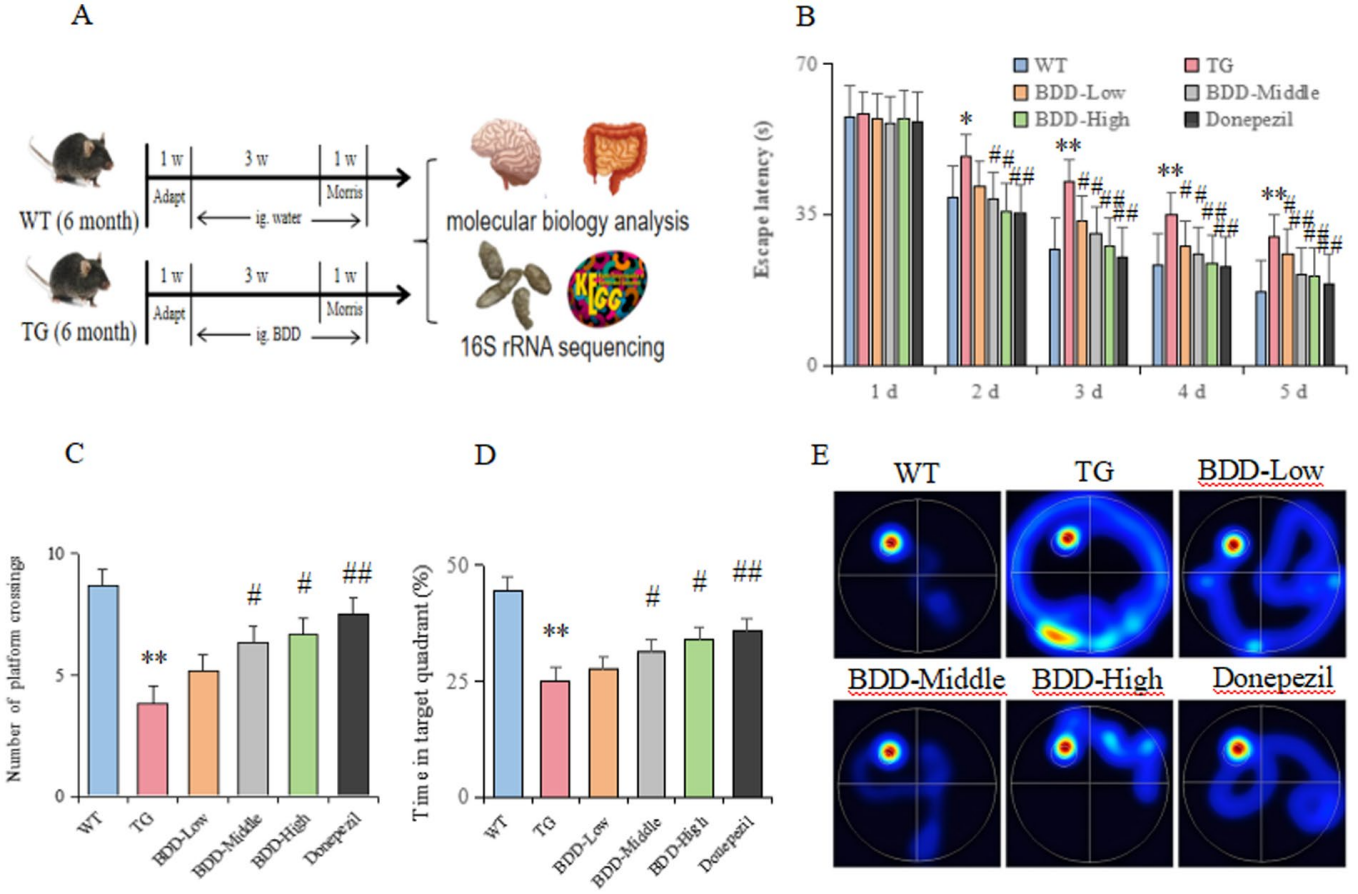
MWM test evaluation of BDD effects on TG mice cognition. **(A)** The time flow chart of mice; **(B)** escape latency; **(C)** platform-crossing frequency; **(D)** target-quadrant dwell time; **(E)** representative swim paths on day 5 of the navigation test. *n* = 10. ^*^p < 0.05, ^**^p < 0.01 vs. WT group: *^#^p* < 0.05, *^##^p* < 0.01 vs. TG group.

As depicted in the experimental timeline (Figure 4A), all groups demonstrated a daily decrease in escape latency. Compared with the TG group, all drug-administered groups showed shortened latencies to varying degrees, with the medium and high-doses BDD groups and the donepezil group exhibiting the most significant improvements (Figure 4B, *p* < 0.05). In the spatial probe test, the TG group crossed the platform location significantly fewer times and spent less time in the target quadrant than the WT group (Figures 4C,D, *p* < 0.01). Conversely, all treatment groups showed marked improvements in these metrics, particularly the medium- and high-dose BDD groups (*p* < 0.01). The results showed that BDD ameliorated spatial learning and memory deficits in TG mice, enhancing cognitive performance with an efficacy comparable to that of donepezil. Since no notable difference was observed between the medium and high doses, a dose of 20 g/kg of BDD was selected for subsequent experiments (Figure 4E).

#### 3.3.2 Effects of BDD on colon and brain injury in TG mice

**Figure 5:**
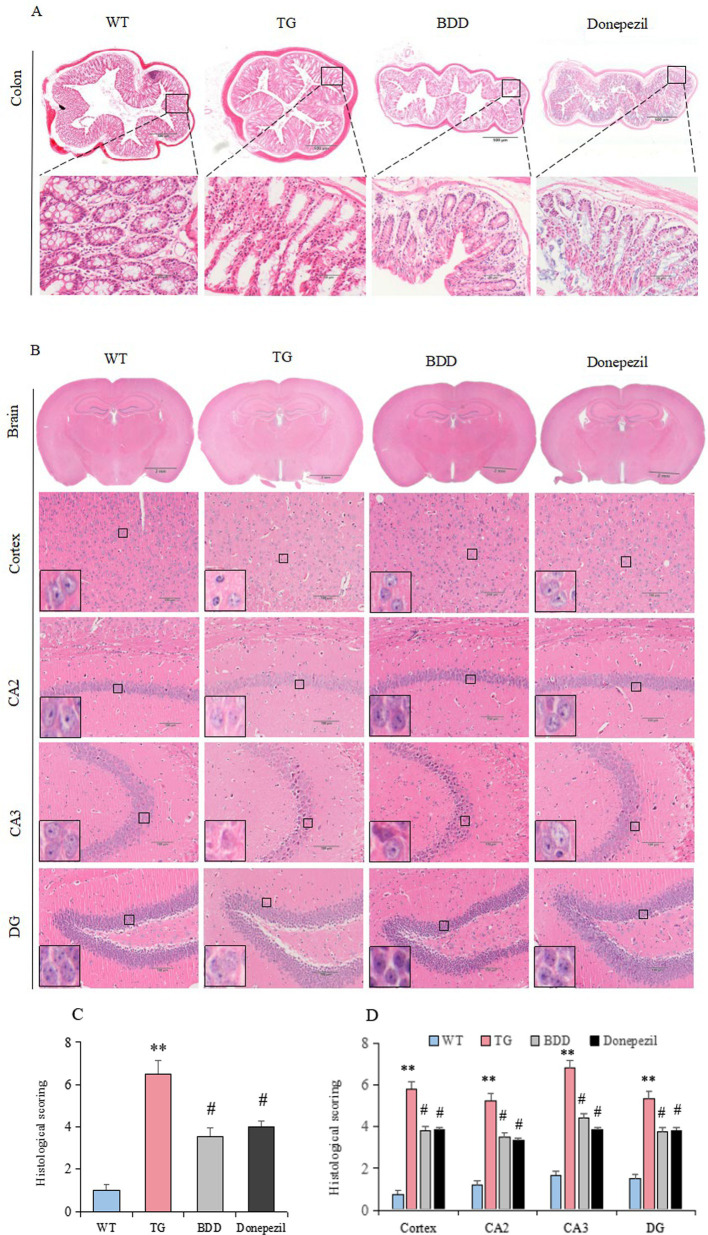
Effects of BDD treatment on the colon and brain function in TG mice. **(A)** Representative photomicrographs of HE-stained colon sections; **(B)** representative photomicrographs of HE-stained brain tissue sections; **(C,D)** histological scoring of colon and brain. *n* = 3. ^**^p < 0.01 vs. WT group; *^#^p* < 0.05 vs. TG group.

Histological analysis of the colon (Figure 5A) displayed that in the WT group, crypts and goblet cells were clearly visible, and the mucosal intestinal epithelial cells and intestinal glands were abundant and neatly arranged without any signs of congestion. In contrast, the colonic crypt architecture in TG mice was severely disrupted, with substantial loss of goblet cells and extensive neutrophil infiltration into the lamina propria, indicating severe ulceration and inflammation. Compared with the TG group, the BDD treatment groups and donepezil group showed less colonic damage, characterized by intact crypts, regular glands, normal epithelium, and fewer infiltrating immune cells (Figure 5C).

Histological analysis of the brain (Figure 5B) showed that in the WT group, nerve cells were abundant with clear layers, abundant cytoplasm, large and round nuclei, and uniformly stained cytoplasm and nuclei, indicating normal tissue morphology and structure. In the TG group, the number of neurons was markedly decreased, with loose and irregular arrangement, deeply stained and constricted nuclei, and obvious nerve cell vacuolation, indicating significant brain tissue damage. The BDD treatment and donepezil groups showed significant improvements in neuronal cell count, cellular layering, and nuclear morphology compared with the TG group (Figure 5D). Specifically, these treatment groups displayed a greater number of neurons, more defined cellular layers, consistent cytoplasmic and nuclear staining, and considerably reduced nuclear shrinkage. These findings indicated that both BDD and donepezil effectively mitigated brain tissue damage in TG mice. Collectively, BDD not only ameliorated colonic tissue injury but also exerted robust neuroprotective effects in TG mice.

#### 3.3.3 Effects of BDD on the ultrastructure of hippocampal neurons in TG mice

**Figure 6:**
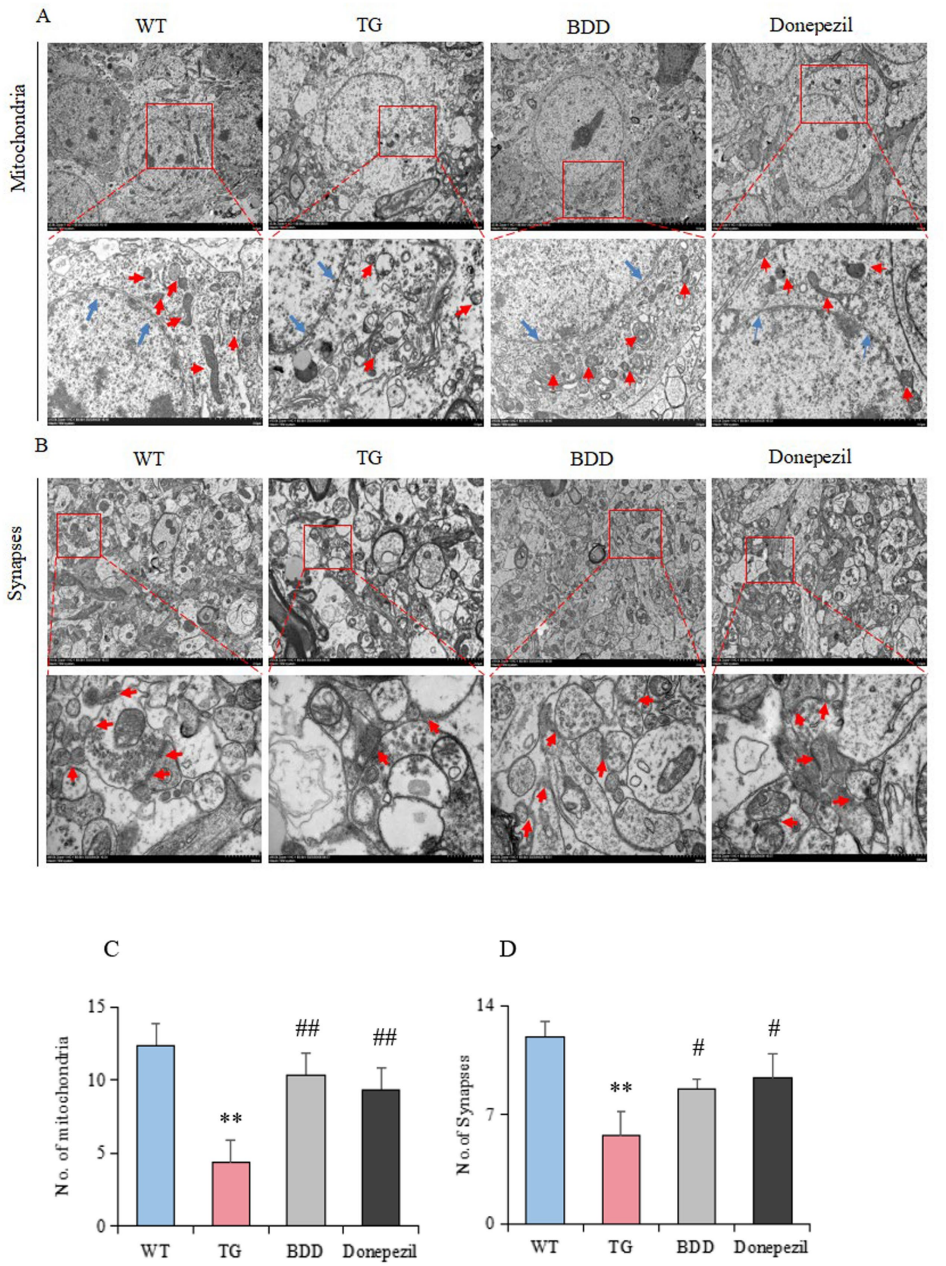
BDD restored neuronal ultrastructural integrity in TG mice. **(A,B)** Representative TEM micrographs of neuronal mitochondria and synapses; **(C,D)** quantitative analysis of mitochondrial counts per group. Scale bar: 500 nm. *n* = 3. ^**^p < 0.01 vs. WT group; ^#^*p* < 0.05, ^##^*p* < 0.01 vs. TG group.

To evaluate whether BDD can ameliorate the ultrastructural deficits of hippocampal neurons in TG mice, ultrathin brain sections were examined by TEM. According to the results, in the WT group, the nuclear membrane of neurons in the CA1 region was smooth (indicated by a long blue arrow), with occasional chromatin condensation; mitochondria were round and with occasional loss of mitochondrial crest (indicated by a short red arrow); and Golgi vesicles were regular. In the TG group, the nuclear membrane appeared folded and ruptured, the nucleus was shrunk, chromatin was condensed, mitochondria were excessively expanded and decreased in number, mitochondrial crest were significantly decreased, and Golgi vesicles were ruptured (Figure 6). In comparison, in the BDD and donepezil groups, neurons in the CA1 region exhibited smooth nuclear membranes with distinct double-layer structures (Figure 6, *p* < 0.05, vs. TG group). Mitochondrial cristae were clearly visible, and Golgi vesicles appeared regular. Furthermore, BDD and donepezil administration notably increased the number of synapses compared with the TG group (Figure 6, *p* < 0.05), along with an elevation in the density of postsynaptic densities. These results indicated that BDD improved mitochondrial and synaptic integrity, which contributed to enhanced learning and memory capacities in TG mice.

#### 3.3.4 Effects of BDD on colonic inflammatory in TG mice

**Figure 7:**
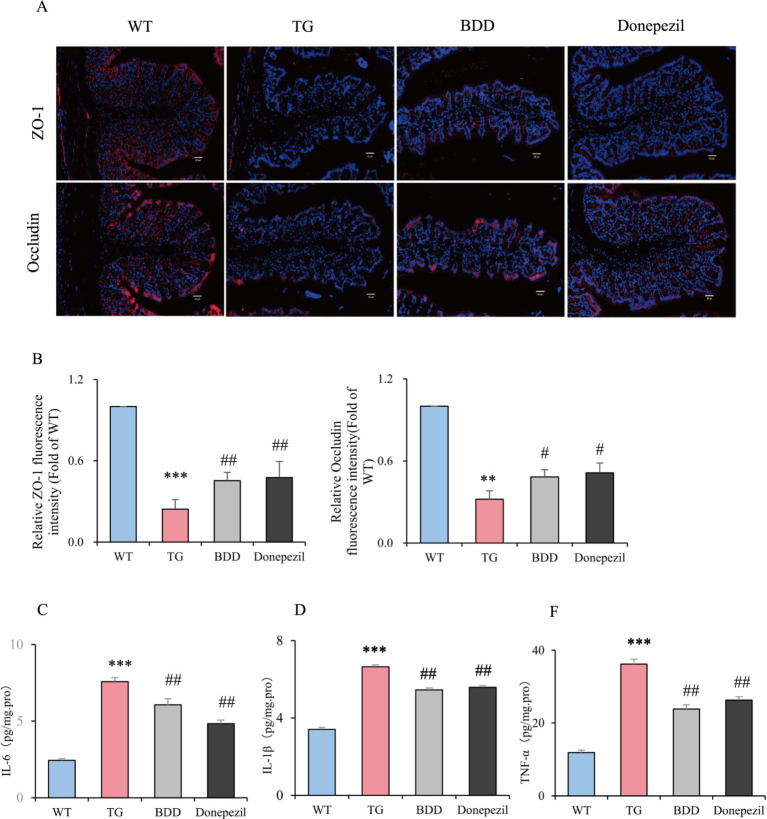
Effects of BDD on pathological damage in each group of mice. **(A,B)** Immunofluorescence and quantification of ZO-1 and Occludin in colon. *n* = 3, Scale bar: 50 μm. **(C–F)** The colonic inflammatory cytokine expression analysis. *n* = 3. ^**^p < 0.01, ^***^p < 0.001 vs. WT group; ^#^*p* < 0.05, ^##^*p* < 0.01 vs. TG group.

In APP/PS1 mice, we evaluated the tight junction protein ZO-1 and Occudin expression in colon from APP/PS1 mice, and it showed the expressions of ZO-1 and Occludin were both down regulated, which were reversed in BDD and donepezil (Figures 7A,B). Subsequently, inflammatory cytokines in each group were profiled using ELISA, the resulet showed that, TG mice demonstrated markedly elevated concentrations of IL-6, IL-1β, and TNF-α compared with WT mice (Figure 7C, *p* < 0.001); both BDD and donepezil treatment effectively lowered these cytokine concentrations (*p* < 0.01). Taken together, BDD attenuated inflammatory expression in the colon, thereby exerting therapeutic effects in TG mice.

#### 3.3.5 Effects of BDD on inflammatory and related factors in the brain

**Figure 8:**
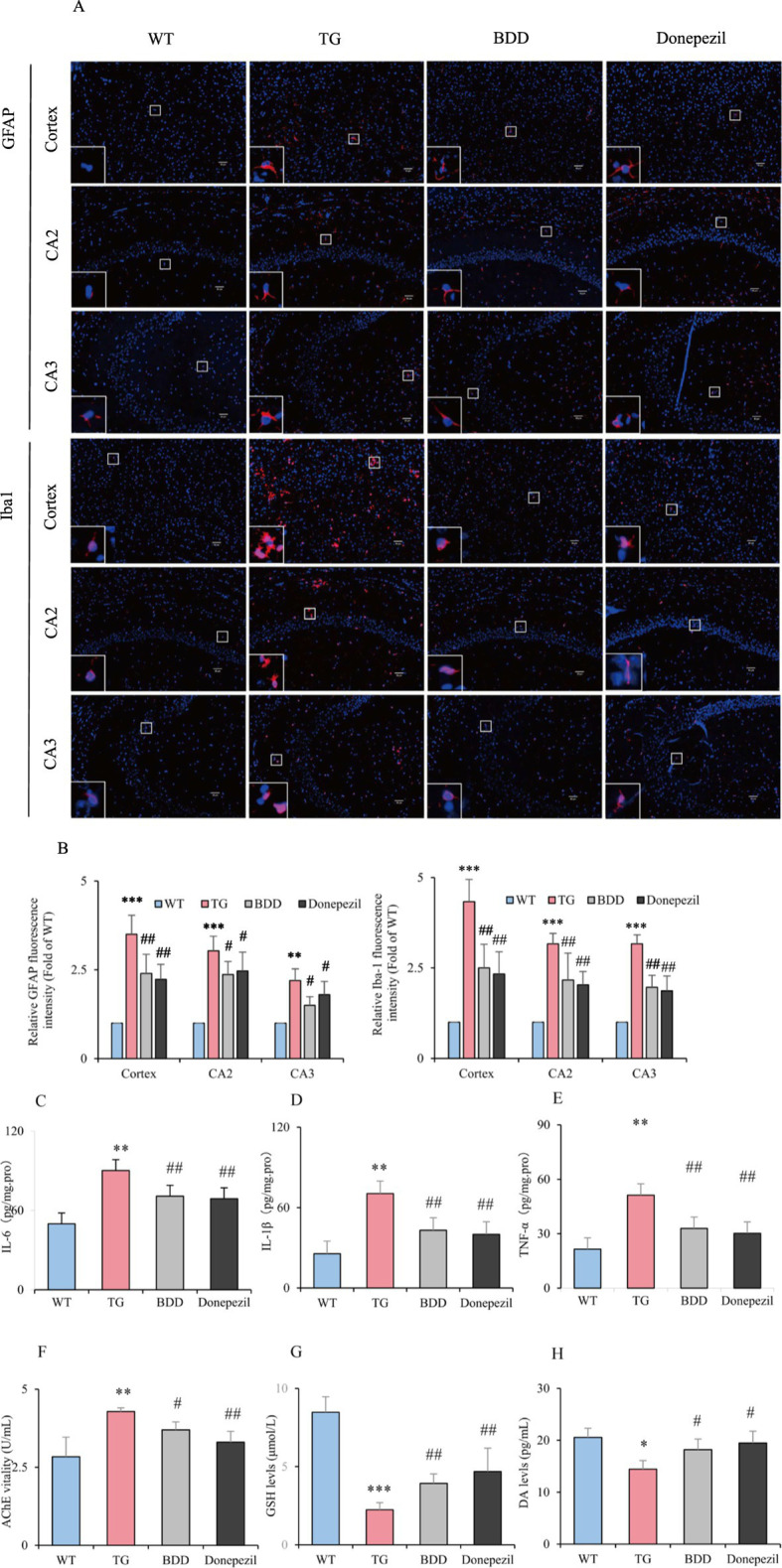
Effects of BDD on neuroactive molecules in TG mouse. **(A,B)** Immunofluorescence and quantification of GFAP and Iba1 in brain. *n* = 3, Scale bar: 50 μm. **(C–E)** Quantitative analysis of inflammatory cytokine in serum; **(F–H)** quantitative analysis of AChE **(D)**, GSH **(E)**, and DA **(F)** in serum. *n* = 3. ^*^p < 0.05, ^**^p < 0.01, ^***^p < 0.001 vs. WT group; ^#^*p* < 0.05, ^##^*p* < 0.01 vs. TG group.

Immunofluorescence labeling was used to detect the astrocytic and microglial activation markers GFAP and Iba1 in the brain to investigate neuroglial activation. As depicted in Figure 8A, the APP/PS1 group exhibited markedly enhanced immunoreactivity for GFAP and Iba1 within the cerebral cortex, CA2 and CA3, indicative of substantial glial activation (Figures 8A,B; *p* < 0.001), both BDD and donepezil reversed these trends, manifested as reduced GFAP and Iba1 levels (*p* < 0.05; *p* < 0.01). Regarding systemic inflammation (Figures 8C–E), serum levels of the pro-inflammatory cytokines IL-1β, IL-6, and TNF-α were significantly elevated in the APP/PS1 group compared to the WT group (*p* < 0.01), indicating an exacerbated peripheral inflammatory response. Conversely, treatment with either BDD or donepezil resulted in a marked reduction in the levels of all three cytokines relative to the APP/PS1 group (*p* < 0.01) This demonstrates that both interventions effectively attenuate systemic inflammation. Moreover, the APP/PS1 group showed elevated AChE level and reduced GSH and DA levels in serum (Figures 8F–H; *p* < 0.01, *p* < 0.001, *p* < 0.05). Both BDD and donepezil reversed these trends, manifested as reduced AChE level and increased GSH and DA levels; BDD outperformed donepezil (Figures 8D–F; BDD: *p* < 0.01; donepezil: *p* < 0.05), underscoring its neuroprotective potential in this AD model.

### 3.4 Effect of BDD in GM

#### 3.4.1 Effects of BDD on the structure of GM in TG mice

**Figure 9:**
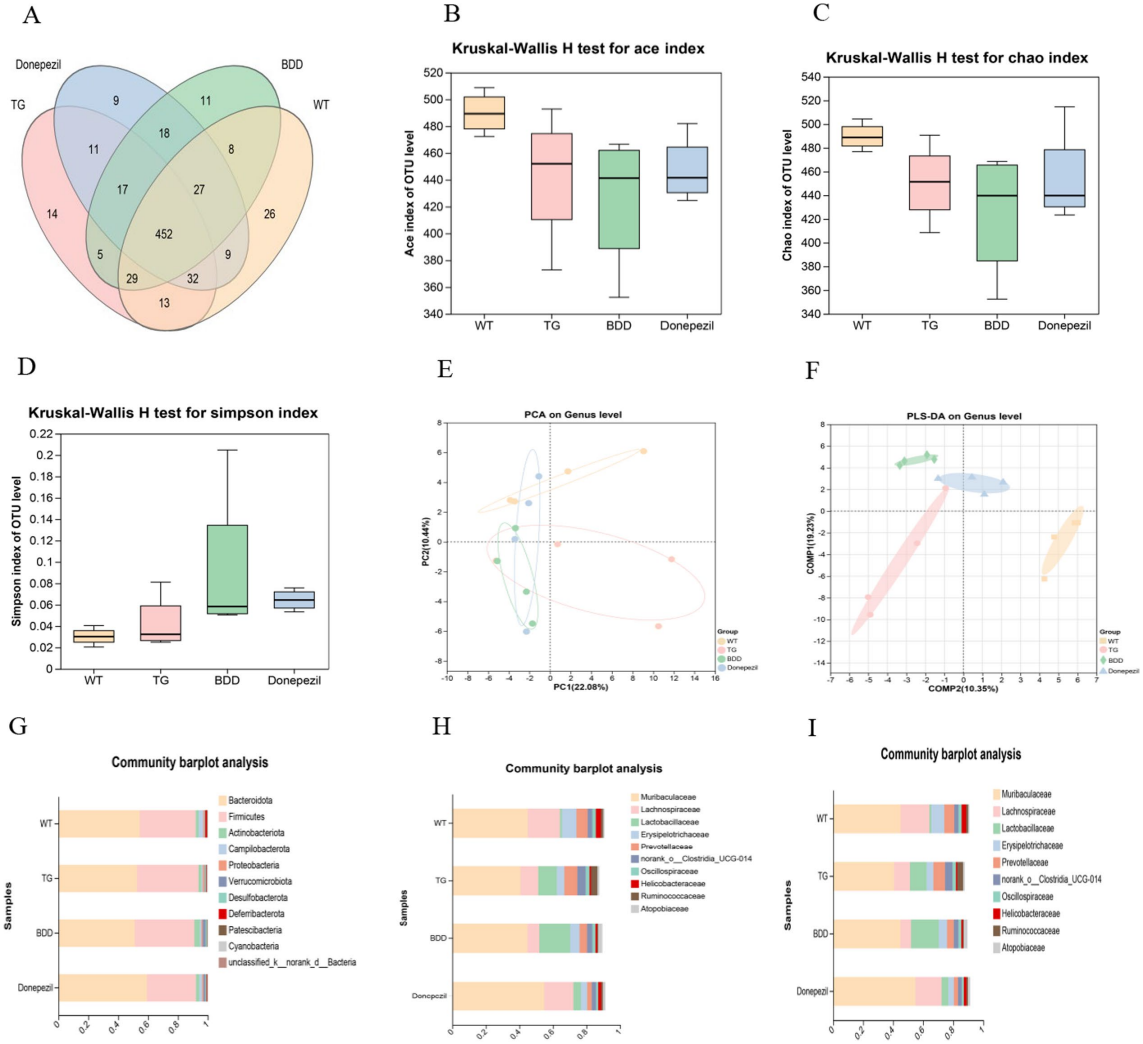
Differences in GM composition among groups. **(A)** Species Venn diagram; **(B–D)** α-diversity indices, including Ace index, Chao index, and Simpson index; **(E)** PCA analysis; **(F)** PLS-DA analysis at the genus level; **(G–I)** colony composition analysis in each group of mice at the phylum level **(G)**, family level **(H)**, and genus level **(I)**. *n* = 4.

Principal coordinate analysis (PCoA)-based analysis was performed to assess the impact of BDD on the GM of TG mice (Pan et al., 2021). A total of 452 OTUs were identified across all groups. The number of unique OTUs in each group was 26 in the WT group, 14 in the TG group, 11 in the BDD group, and 9 in the donepezil group (Figure 9A). As shown by the ACE and Chao indices (Figures 9B,C), BDD and donepezil did not increase the total count of GM in TG mice. However, the Simpson index (Figure 9D) revealed that BDD significantly improved the richness of the GM. According to PCoA analysis results (Figure 9E), at the OTU level, the WT and TG groups were well separated, while the BDD and donepezil groups had partial overlap. The results indicated marked divergence in community structure across groups. Furthermore, partial least squares-discriminant analysis (PLS-DA) results revealed that the sample clusters of the WT and TG groups occupied distinct regions, whereas those of the BDD and donepezil groups were situated between them, further indicating distinct microbial community structures across the groups (Figure 9F). Collectively, these indexes showed that although BDD and donepezil did not increase the overall abundance of GM, they enhanced microbial diversity by reducing the abundance of certain microflora.

Additionally, changes in the composition of intestinal flora were assessed at different taxonomic levels. At the family level, BDD increased the abundance of *Lactobacillaceae* and *Oscillospiraceae*, while decreasing that of *Erysipelotrichaceae*, *Prevotellaceae*, and *norank_o__Clostridia_UCG-014* (Figure 9G). Furthermore, at the genus level, BDD treatment resulted in a decrease in the abundance of *norank_f__Muribaculaceae*, *Lachnospiraceae_NK4A136_group*, *Dubosiella*, *Prevotellaceae_UCG-001*, *norank_f__norank_o__Clostridia_UCG-014*, and *unclassified_f__Lachnospiraceae*, alongside an increase in the abundance of *Lactobacillus* and *Coriobacteriaceae_UCG-002* (Figures 9H,I). In conclusion, these results demonstrated that BDD treatment induced significant alterations in the GM structure of TG mice.

#### 3.4.2 Typing analysis of GM and functional prediction

**Figure 10:**
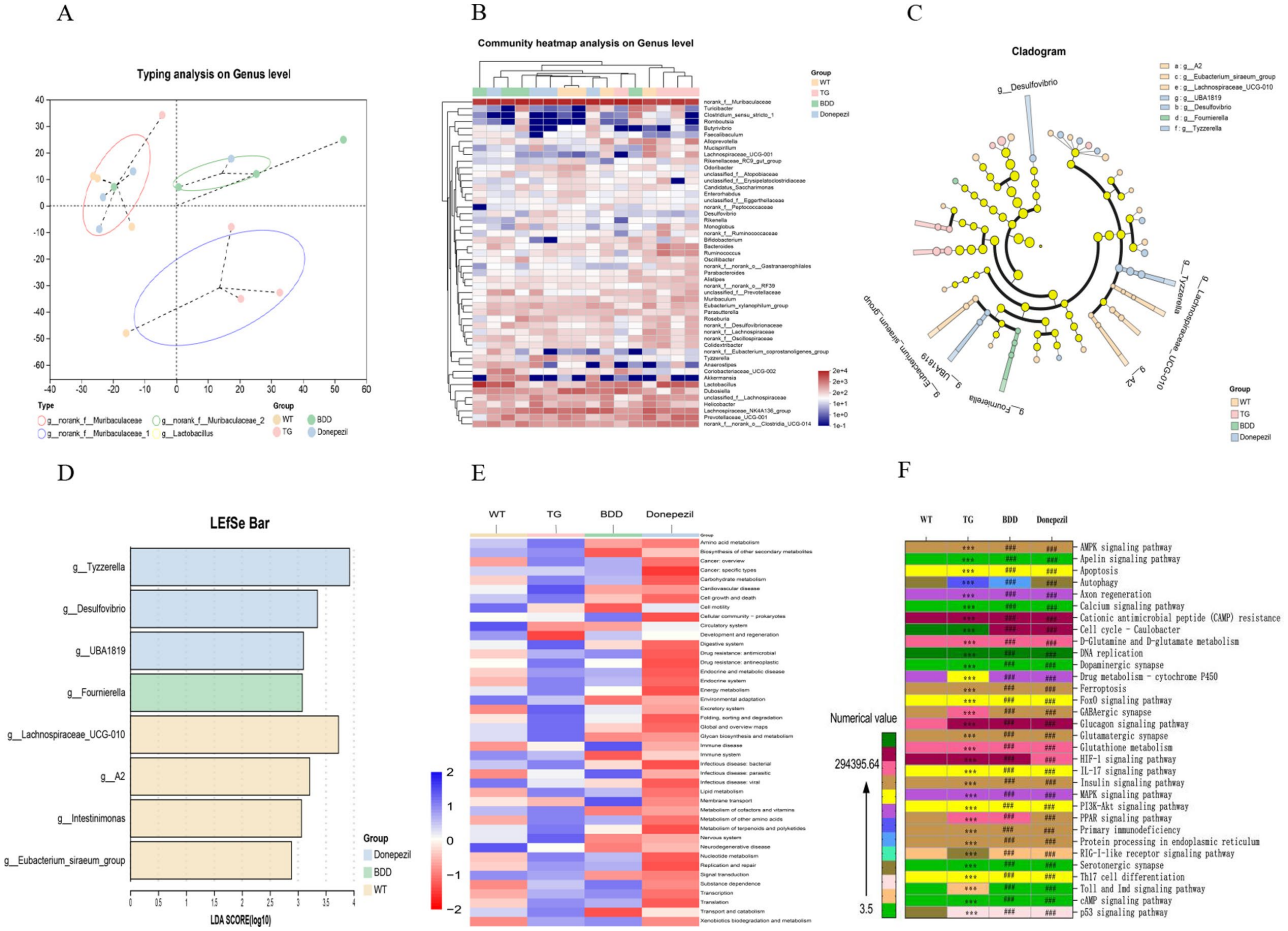
Difference analysis of GM and signaling pathways among groups. **(A)** Typing analysis of sample flora; **(B)** Clustering analysis results; **(C)** taxonomic tree of the dominant flora; **(D)** microbiota dominance analysis; **(E)** PICRUSt2 functional prediction analysis; **(F)** difference analysis of signaling pathways. *n* = 4. ^***^p < 0.001 vs. WT group; ^###^*p* < 0.001, vs. TG group.

The results indicated that the WT, BDD, and donepezil groups shared similar dominant species: *Muribaculaceae*, *Lactobacillaceae*, and *Lactobacillus*. Conversely, the TG group demonstrated distinct clustering, particularly evident at the genus level (Figure 10A). Subsequently, a cluster analysis based on species abundance similarity was conducted. The results revealed that at the genus level, the BDD and donepezil groups tended to cluster together (Figure 10B). This suggested that both BDD and donepezil can modulate the intestinal microorganism composition in TG mice, resembling the microflora profile of the WT group. Furthermore, a taxonomic tree of dominant groups was constructed and visualized (Figures 10C,D). The analysis identified that the prevalent bacteria in the WT group included *g_A2, g_Eubacterium_siraeum_group*, and *grub Lachnospiraceae UCG LCG*. In contrast, the dominant bacteria in the donepezil group were *g_UBA1819*, *g_Desulfovibrio*, and *g_Tyzzerella*. Consequently, these results indicated that BDD may enhance cognitive function by modulating the intestinal flora composition in TG mice.

Microbial functions were analyzed using PICRUSt2 software, with sequencing data compared and predicted against the KEGG database (Zhu et al., 2024). The analysis identified 285 interconnected signaling pathways in total. After excluding low-expression functional pathways, 43 KEGG L2 and 114 KEGG L3 pathways showed significant differences among the three groups. KEGG L2 functional pathways include NDs, the immune system, the nervous system, signal transduction, and other related categories. A total of 32 KEGG L3 pathways were identified as differentially regulated, encompassing the AMPK, apoptosis, autophagy, FoxO, HIF-1, IL-17, PI3K-Akt, MAPK, cAMP, and p53 signaling pathways. According to the analysis results of the difference parameter values of each group (Figures 10E,F), the signal pathways related to the inflammatory syndrome in the TG group (*vs.* WT group, *p* < 0.001), while the parameter values in the BDD and donepezil groups were similar to those in WT littermates, showing statistically significant difference (*p* < 0.001). Therefore, we hypothesized that BDD improved cognition via the GBA.

### 3.5 Molecular docking and dynamics simulation analysis

**Figure 11:**
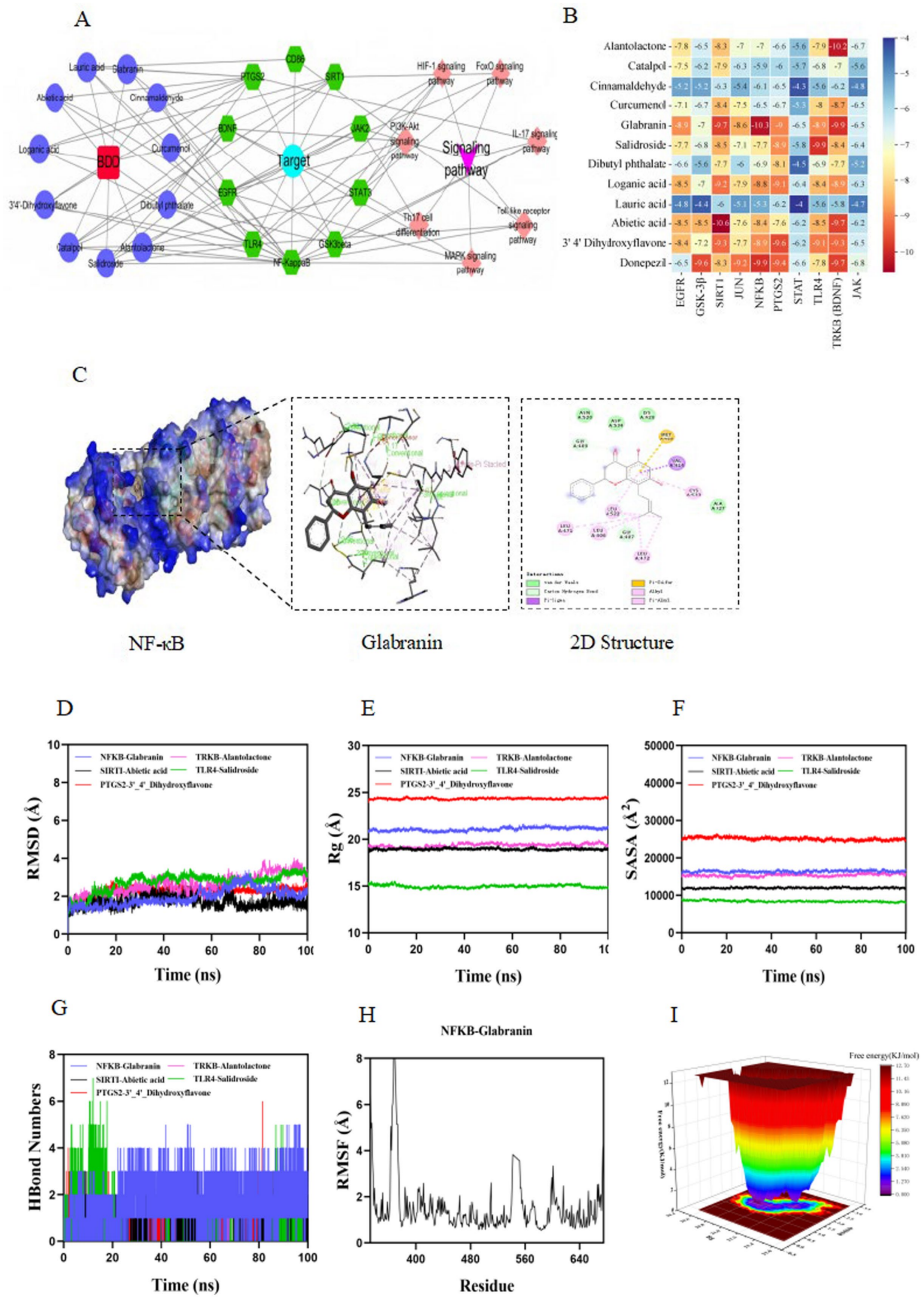
Visualization of molecular docking. **(A)** Integration of network pharmacology with GM enrichment analysis; **(B)** molecular docking scores; **(C)** Visualization of core targets (representative figure); **(D)** RMSD values of the complexes; **(E)** radius of gyration (Rg) values of the complexes; **(F)** SASA values of the complexes; **(G)** hydrogen bond numbers of the complexes; **(H)** RMSF values of the complexes (representative figure); **(I)** free-energy landscape (representative figure).

Through integrating network pharmacology with GM enrichment analysis, 7 common signaling pathways were identified: FoxO, MAPK, PI3K-Akt, HIF-1, Th17, IL-17, and Toll/Imd signaling pathways. The associated targets included TLR4, PTGS2, SIRT1, BDNF, NF-κB, STAT3, JAK2, EGFR, GSK3β, and CD86. These targets were primarily associated with compounds such as shanzhiside, catalpol, salidroside, and abietic acid (Figure 11A).

The selected core targets were docked with the top 11 core components (those comprising more than 2 nodes). A docking score below −5.0 kJ/mol was commonly considered indicative of strong affinity with the target (Shi et al., 2025). Molecular docking was performed using AutoDockTools, yielding 120 docking outcomes (Figure 11B). Among them, 113 groups demonstrated binding energies below −5.0 kJ/mol (Figure 11B), suggesting spontaneous binding potential of the compounds. Subsequently, the optimal docking configurations of 10 core targets were visualized using Discovery Studio software, as depicted in Figure 11C.

The equilibrium of the simulation systems was evaluated using root mean square deviation (RMSD). The five complexes with the lowest docking binding energies were subjected to molecular dynamics simulations to assess their stability. As shown in Figures 11D–I, the TLR4-salidroside, NFKB-glabranin, TRKB-alantolactone, PTGS2-3′, 4′-dihydroxyflavone, and SIRT1-abietic acid complexes fluctuated below 3 Å throughout the entire trajectory (Figure 11D). No significant expansion or contraction was observed (Figure 11E). The solvent accessible surface area (SASA) values remained essentially unchanged (Figure 11F), the complexes exhibited favorable hydrogen-bond interactions (Figure 11G), and the root mean square fluctuation (RMSF) values of each complex stayed low (Figures 11H,I). These results indicated that the complexes bound stably and maintained robust hydrogen-bond networks. Consequently, salidroside, glabranin, alantolactone, 3′,4′-dihydroxyflavone, and abietic acid exhibited favorable binding to the TLR4, NFKB, SIRT1, TRKB, and PTGS2 target proteins, respectively.

### 3.6 QSAR and WB analysis

**Figure 12:**
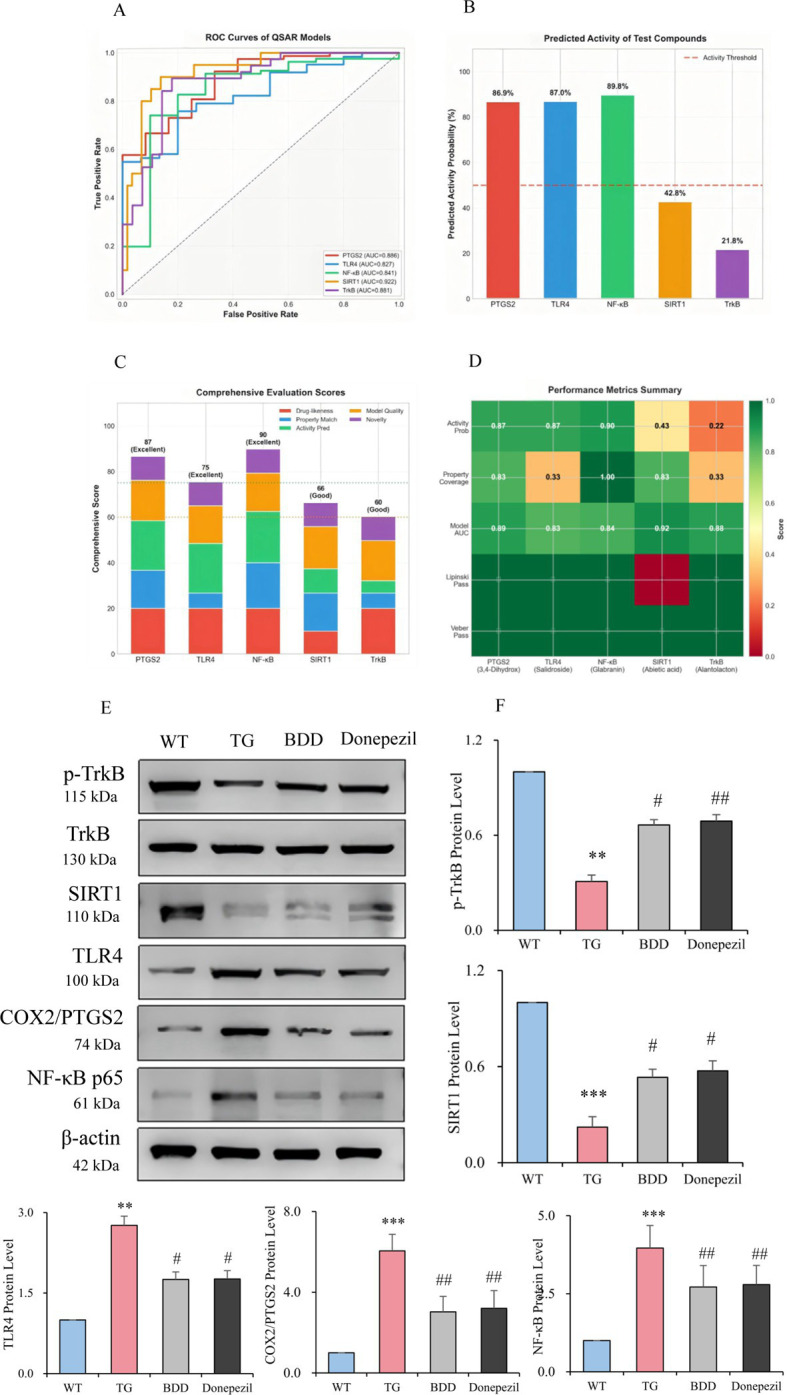
Effects of BDD on hippocampal related proteins in each group. **(A)** ROC curves of the five QSAR models; **(B)** activity probability prediction of active ingredients-targets (the dashed line indicates the 50% activity threshold); **(C)** breakdown of composite scores; **(D)** heatmap of performance metrics. Green indicates high scores/passing; red indicates low scores/failure; **(E)** representative WB images; **(F)** densitometric analysis of protein bands. *n* = 3. ^**^p < 0.01, ^***^p < 0.001 vs. WT group; ^#^*p* < 0.05, ^##^*p* < 0.01, vs. TG group.

QSAR analysis results showed that the ROC curves of all five models were positioned in the upper-left region of the diagonal, with AUC values ranging from 0.827 to 0.922. This indicated that the established models possessed a reliable capability to distinguish between active and inactive compounds (Figure 12A). Activity prediction revealed that glabranin exhibited the highest probability of activity against the NF-κB target at 89.8%, followed by salidroside-TLR4 at 87.0%, 3′,4′-dihydroxyflavone-PTGS2 at 86.9%, and abietic acid-SIRT1 at 42.8%, while alantolactone-TRKB displayed the lowest activity at 21.8% (Figure 12B). Although this value fell below the activity threshold, it remained reasonable considering the structural differences between natural products and synthetic drugs, as well as the fact that the QSAR models were primarily constructed based on synthetic compound datasets. Furthermore, a quantitative assessment integrating five dimensions (drug-likeness, chemical space matching degree, activity prediction confidence, model reliability, and structural novelty) demonstrated that the comprehensive scores of the five compounds ranged between 60 and 90 points. Among these, glabranin (90 points), 3′,4′-dihydroxyflavone (87 points), and salidroside (75 points) received an “Excellent” rating, whereas abietic acid (66 points) and alantolactone (60 points) were rated as “Good” (Figure 12C). The performance metric heatmap indicated that glabranin, 3′,4′-dihydroxyflavone, salidroside, and alantolactone all passed the screening criteria of Lipinski’s Rule of Five and Veber’s rules, suggesting favorable potential for oral bioavailability (Figure 12D).

Subsequently, Western blotting (WB) analysis was performed to detect the expression of the five aforementioned proteins to further validate the predictive results. It was found that, compared with the control (WT) group, hippocampal expression levels of SIRT1 and p-TrkB proteins were notably decreased, whereas expression levels of TLR4, NF-κB p65, and COX2/PTGS2 proteins were remarkably elevated in the TG group (Figures 12E,F; *p* < 0.01, *p* < 0.001). Conversely, in mice administered BDD or donepezil, SIRT1 and p-TrkB expressions were significantly upregulated, while TLR4, NF-κB p65, and COX2/PTGS2 expressions were notably downregulated compared with mice in the TG group (Figures 12E,F; *p* < 0.05, *p* < 0.01). These findings corroborated the prediction results and indicated that BDD exerted therapeutic effects against AD through a multi-component, multi-target mechanism, with efficacy comparable to that of donepezil.

## 4 Discussion

**Figure 13:**
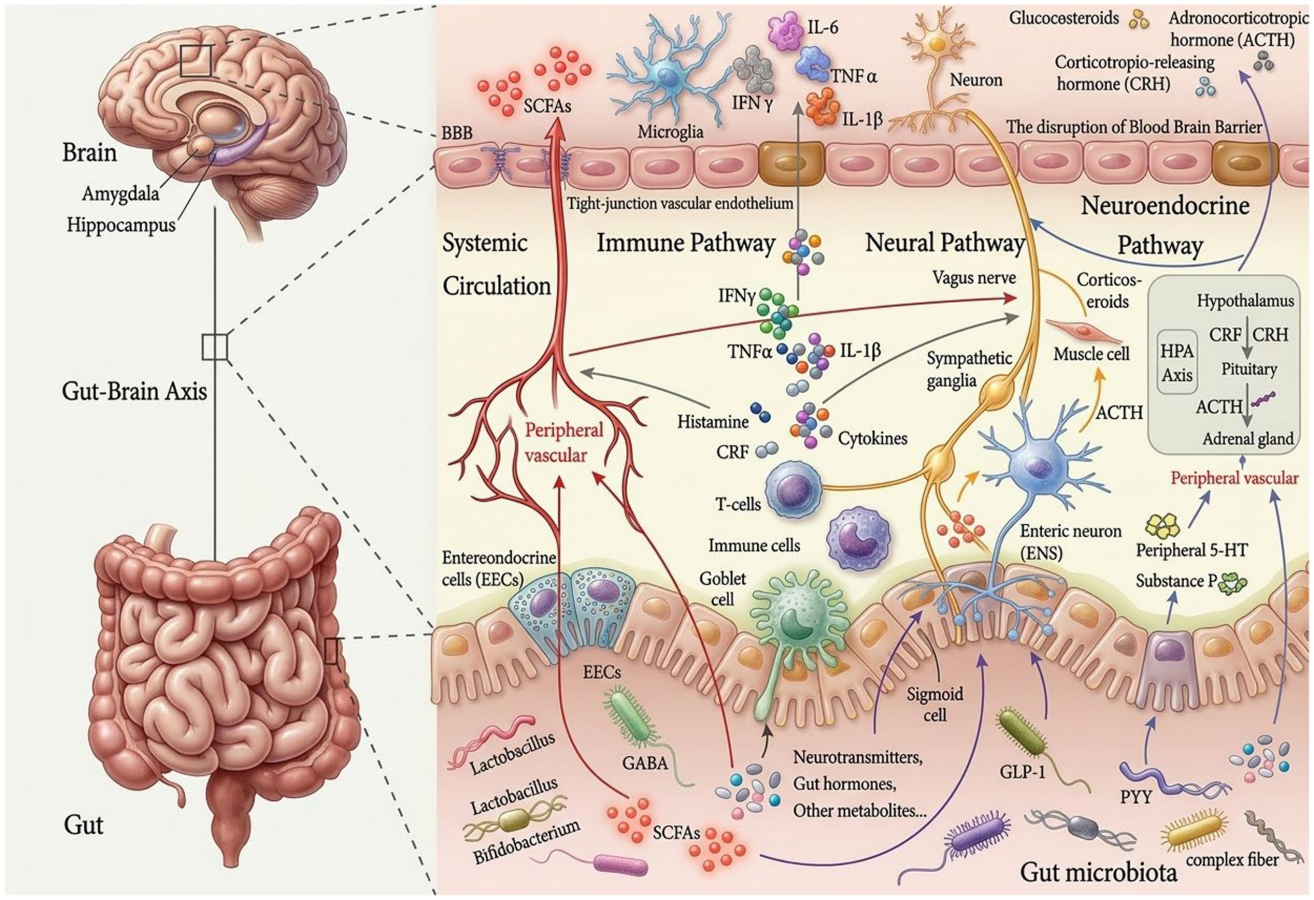
Schematic diagram of AD pathology in the GBA (Wang et al., 2025). Various communication pathways of the GBA, such as neural (e.g., vagus nerve), immune (e.g., cytokines), and neuroendocrine pathways (e.g., gut hormone secretion), were influenced by GM. These pathways involved both direct (e.g., enteric nervous system and vagus nerve) and indirect (e.g., neurotransmitters) connections.

With further elucidation of the mechanism linking the GM and the CNS, it is clear that GM serves as a key regulator of immune cells in the GBA. The GM not only influences host immune responses through its metabolites but also modulates the function of peripheral immune cells, thereby affecting the brain’s inflammatory status in AD patients. Accumulating evidence has indicated that gut dysbiosis is closely associated with impaired BBB integrity and enhanced neuroinflammation. Structural alterations in the GM lead to aberrant stress responses, altered neurotransmitter levels, and cognitive dysfunction. This further emphasizes the complex microbiota-immune-neural interplay (Wang et al., 2025). Therefore, regulating GM to prevent and treat AD has merged as a new research focus (Figure 13) (Liu et al., 2020; Mou et al., 2022; Wang et al., 2025). The Chinese herbal formula BDD is widely acknowledged for its efficacy in addressing mental health conditions (such as mental instability, absentmindedness, insomnia, and deficient dysphoria), which are symptoms typically associated with AD development. Originating from the ancient text “*Jinkui Yaolue*” by Zhang Zhongjing in the early 3rd century, BDD comprises two key herbs: Baihe (*Lilii Bulbus*) and Dihuang (*Rehmanniae Radix*). With a long history of clinical application spanning millennia in China, BDD presents a promising potential alternative for the treatment of AD.

In the present study, we adopted an integrated strategy combining serum pharmacokinetics with network pharmacology to elucidate the pharmacodynamic basis and mechanistic underpinnings of the cognitive benefits of BDD. Initially, 49 components of BDD were detected in serum, primarily consisting of phenylpropanoids, lipids, organic acids, and alkaloids. Through analysis of multiple databases, 118 targets associated with AD were identified. A PPI network was then constructed, and the top 13 targets with the highest degree values were selected: TLR4, PTGS2, AKT, SIRT1, BDNF, MPO, NF-κB, STAT3, JAK2, EGFR, CXCR4, GSK3β, and CD86. Further GO enrichment analysis revealed that BDD may influence BPs, CC, and MFs through multiple pathways, thereby participating in the regulation of pathophysiological changes in AD. KEGG enrichment analysis revealed multiple AD-associated signaling pathways, such as PI3K-Akt, FoxO, Th17, JAK–STAT, IL-17, MAPK, NF-κB, and EGFR. Recent studies have shown that neuroimmune and inflammation are common pathophysiological features of all CNS disorders (Figure 3). When dysregulated, overactivated, or persistently present, neuroimmune responses can exacerbate neuronal injury, forming a vicious cycle of “neuroinflammation-neurodegeneration” (Mou et al., 2022). The signaling pathways identified in this study are all associated with neuroinflammation. Modulating these pathways may alleviate AD-related pathological damage, suppress the release of inflammatory factors, and reduce neuronal loss, thereby slowing AD progression (Gouveia et al., 2024; Kumari et al., 2025). Based on these findings, we hypothesized that BDD may exert its therapeutic effects against AD primarily through inhibiting neuroinflammation.

In this study, inflammatory damage in TG mice was examined using a series of assays (MWM test, HE staining, TEM test, and ELISA) to evaluate the anti-inflammatory effects of BDD. Our findings indicated that BDD enhanced learning and memory in TG mice while reducing the expression of inflammatory factors in both colon and brain tissues. These effects were evidenced by significantly increased cell numbers within colon and brain tissues, reduced nuclear pyknosis, and increased numbers of mitochondria and synapses in the brain, with outcomes comparable to those of donepezil. Notably, it has been shown that damage to both colon and brain tissues increases intestinal permeability and compromises the BBB, which can trigger peripheral and CNS inflammation and ultimately lead to neurological disorders (Kang et al., 2024). Furthermore, GM dysbiosis has been shown to increase intestinal mucosa and BBB permeability by promoting pro-inflammatory microbial flora and bacteria-derived LPS, thereby compromising barrier integrity and contributing to the onset of AD (Varesi et al., 2022; Duggan et al., 2025).

Next, GM abundance in each group of mice was analyzed using 16 s rRNA sequencing. The results showed that *Bacteroides* and *Firmicutes* were the dominant bacteria in all groups. According to α-diversity analysis results, BDD did not increase the total number of intestinal microflora species but notably improve the richness index of the GM in TG mice. On the other hand, β-diversity analysis results showed that BDD and donepezil effectively restored the microbial community structure in TG mice. These findings suggested that BDD decreased the prevalence of specific dominant bacteria and enhanced GM diversity in TG mice, thereby helping to re-establish a proper intestinal flora structure. At the family level, BDD elevated the relative abundance of *Lactobacillaceae* and *Oscillospiraceae* while reducing that of *Erysipelotrichaceae*, *Prevotellaceae,* and *norank_o__Clostridia_UCG-014*. At the genus level, BDD decreased the relative abundance of *Prevotellaceae_UCG-001, Dubosiella, norank_f__Muribaculaceae, unclassified_f__Lachnospiraceae, norank_f__norank_o__Clostridia_UCG-014,* and *Lachnospiraceae_NK4A136_group,* and increased the abundance of *Coriobacteriaceae_UCG-002 and Lactobacillus*. As has been evidenced previously, *Lactobacillaceae (Lactobacillus)* acts as a probiotic and contributes substantially to the treatment of NDs. It regulates the metabolism of key substances (such as tryptophan), promotes beneficial metabolites (including neurotransmitters), and reduces both intestinal permeability and inflammation (Liu et al., 2020; Mayer et al., 2022). Additionally, *Erysipelotrichaceae, Prevotellaceae,* and *norank_o__Clostridia_UCG-014* have all been demonstrated to be related to inflammation. For example, *Erysipelotrichaceae* is present in mammals, and certain strains are pathogenic, capable of causing inflammation and erysipelas. *Prevotellaceae* can reduce IL-18 production, aggravate intestinal inflammation, and contribute to systemic autoimmunity (Bauerl et al., 2018; Sun et al., 2020). *Norank_o__Clostridia_UCG-014* is positively correlated with IL-17, TNF-α (Marizzoni et al., 2023). Therefore, BDD may improve learning and memory in TG mice by modulating the GM to attenuate neuroinflammation.

To further elucidate the mechanism of BDD in ameliorating GM structure and exerting therapeutic effects against AD, this study comprehensively integrated the core mechanisms identified by network pharmacology and PICRUSt2 analysis. A total of 7 common signaling pathways were identified, including FoxO, HIF-1, IL-17, MAPK, PI3K-Akt, Th17, and Toll/Imd. In addition, 11 active components of BDD (3′_4’_dihydroxyflavone, alantolactone, catalpol, cinnamaldehyde, curcumenol, glabranin, salidroside, dibutyl phthalate, loganic acid, lauric acid, abietic acid) and 10 core targets (TLR4, PTGS2, SIRT1, BDNF, NF-κB, STAT3, JAK2, EGFR, GSK3β, and CD86) were identified. These screening results also reflected the multi-component and multi-target characteristics of TCM. Molecular docking revealed that 103 out of 110 binding energy scores between the above components and targets were below −5 kcal/mol, indicating that they theoretically possessed the ability to modulate these signaling pathways and may thereby participate in GBA-related biological processes. Molecular dynamics simulations of the complex conformations of the top five targets and components showed that the interacting pairs—salidroside-PTGS2, alantolactone-TrkB (BDNF), abietic acid-SIRT1, and glabranin-NF-κB—exhibited favorable characteristics in terms of overall protein structure, internal conformation, and interaction strength. Furthermore, QSAR and WB results demonstrated that BDD ameliorated neuroinflammation by regulating the expression of TLR4, PTGS2, SIRT1, TrkB (BDNF), and NF-κB proteins in the brain. These findings are mutually corroborated by the previous molecular docking affinity analysis and molecular dynamics simulation stability assessment, providing multi-level and multi-dimensional validation. Collectively, these results suggested that BDD exerted its therapeutic effects against AD via the GBA.

Nevertheless, this study has several limitations. First, the GM characteristics were assessed in 6-month-old TG mice without longitudinal comparison. Second, the relatively small number of sequencing samples per group may have introduced instability into the assessment of microbial abundance. Most importantly, drug metabolites were not analyzed in this study, and the specific strains responsible for modulating functional microbiome components remain to be identified. Future studies should address these limitations through more in-depth investigation.

## 5 Conclusion

This study integrates serum pharmacochemistry, network pharmacology, molecular docking and dynamics simulation, and *in-vivo* experimentation into a closed-loop workflow: “data-driven hypothesis-experimental validation- computational prediction”. The present study clarifies how BDD ameliorates AD-related cognitive deficits through multi-compounds (e.g., salidroside, glabranin, abietic acid, 3′_4′_dihydroxyflavone, and alantolactone) acting on multi-targets (TLR4, NF-κB, PTGS2, SIRT1, and TrkB) to modulate inflammation-associated pathways. Furthermore, this study offers a transferable paradigm for dissecting complex TCM formulas. Starting from serum-active compounds, network pharmacology pinpointed core pathways and targets; multidimensional phenotyping in animal models provided functional proof; and computational simulations further dissected pivotal GBA interactions. This closed-loop strategy effectively bridges traditional experience with modern science, substantiating the TCM-based “GBA” theory. Future work should prioritize clinical translation (e.g., randomized controlled trials), advanced models (e.g., organoids), cutting-edge technologies (e.g., multi-omics integration, targeted delivery), and systematic evaluation of BDD in AD prevention and treatment, ultimately delivering precision TCM solutions for AD.

## Data Availability

The data presented in the study are deposited in the NCBI Sequence Read Archive (SRA) repository, accession number: PRJNA1480963. Additional data can be found in the Figshare data repository, doi: 10.6084/m9.figshare.32725812. Further inquiries can be directed to the corresponding author.

## Footnotes

1
https://pubmed.ncbi.nlm.nih.gov/

2
https://www.tcmsp-e.com/

3
https://ctdbase.org/

4
https://string-db.org/

5
https://www.rcsb.org/

## Glossary

BDD: Baihe Dihuang decoction
AD: Alzheimer’s disease
GM: Gut microbiota
GBA: Gut-brain axis
TG mice: APP/PS1 double transgenic mice
WT mice: Wild-type mice
IL-6: Interleukin-6
IL-1β: Interleukin-1β
TNF-α: Tumor necrosis factor-α
AChE: Acetylcholinesterase
GSH: Glutathione
DA: Dopamine
KEGG: Kyoto encyclopedia of genes of genomes
GO: Gene ontology
TCM: Traditional Chinese medicine
TCMSP: Traditional Chinese medicine systems pharmacology database and analysis platform
CTD: Comparative toxicogenomics database
16S rRNA: 16S ribosomal RNA sequencing
HE: Hematoxylin and eosin
TEM: Transmission electron microscope
TLR4: Toll-like receptor 4
PTGS2: Prostaglandin-endoperoxide synthase 2
SIRT1: Sirtuin 1
BDNF: Brain-derived neurotrophic factor
NF-κB: Nuclear factor kappa-B
STAT3: Signal transducer and activator of transcription 3
JAK2: Janus kinase-2
EGFR: Epidermal growth factor receptor
GSK3β: Glycogen synthase kinase-3
CD86: Cluster of differentiation 86

## References

[ref1] Bauerl C. Collado M. C. Diaz Cuevas A. Vina J. Perez Martinez G. (2018). Shifts in gut microbiota composition in an APP/PSS1 transgenic mouse model of Alzheimer's disease during lifespan. Lett. Appl. Microbiol. 66, 464–471. doi: 10.1111/lam.12882, 29575030

[ref3] Doifode T. Giridharan V. V. Generoso J. S. Bhatti G. Collodel A. Schulz P. E. . (2021). The impact of the microbiota-gut-brain axis on Alzheimer's disease pathophysiology. Pharmacol. Res. 164:105314. doi: 10.1016/j.phrs.2020.105314, 33246175

[ref4] Duggan M. R. Morgan D. G. Price B. R. Rajbanshi B. Martin-Pena A. Tansey M. G. . (2025). Immune modulation to treat Alzheimer's disease. Mol. Neurodegener. 20:39. doi: 10.1186/s13024-025-00828-x, 40165251 PMC11956194

[ref5] Gouveia F. Fonseca C. Silva A. Camins A. Teresa C. M. Ettcheto M. . (2024). Intranasal irbesartan reverts cognitive decline and activates the PI3K/AKT pathway in an LPS-induced neuroinflammation mice model. Int. Immunopharmacol. 128:111471. doi: 10.1016/j.intimp.2023.111471, 38199198

[ref6] Hardinsyah H. Gunawan W. B. Nurkolis F. Alisaputra D. Kurniawan R. Mayulu N. . (2023). Antiobesity potential of major metabolites from *Clitoria ternatea* kombucha: untargeted metabolomic profiling and molecular docking simulations. Curr Res Food Sci 6:100464. doi: 10.1016/j.crfs.2023.100464, 36875892 PMC9976213

[ref7] Hou J. Wang X. Zhang J. Shen Z. Li X. Yang Y. (2024). Chuanxiong Renshen decoction inhibits Alzheimer’s disease Neuroinflammation by regulating PPARγ/NF-κB pathway. Drug Des. Devel. Ther. 18, 3209–3232. doi: 10.2147/dddt.S462266, 39071817 PMC11283787

[ref8] Kang J. W. Vemuganti V. Kuehn J. F. Ulland T. K. Rey F. E. Bendlin B. B. (2024). Gut microbial metabolism in Alzheimer's disease and related dementias. Neurotherapeutics 21:e00470. doi: 10.1016/j.neurot.2024.e00470, 39462700 PMC11585892

[ref9] Kim S. Chun H. Kim Y. Kim Y. Park U. Chu J. . (2024). Astrocytic autophagy plasticity modulates Abeta clearance and cognitive function in Alzheimer's disease. Mol. Neurodegener. 19:55. doi: 10.1186/s13024-024-00740-w, 39044253 PMC11267931

[ref10] Kim N. Jeon S. H. Ju I. G. Gee M. S. Do J. Oh M. S. . (2021). Transplantation of gut microbiota derived from Alzheimer's disease mouse model impairs memory function and neurogenesis in C57BL/6 mice. Brain Behav. Immun. 98, 357–365. doi: 10.1016/j.bbi.2021.09.002, 34500036

[ref11] Kim M. G. Ooi S. L. Kim G. W. Pak S. C. Koo B. S. (2023). Effectiveness and safety of pattern identification-based herbal medicine for Alzheimer's disease: a systematic review and Meta-analysis. J Integr Complement Med 29, 605–620. doi: 10.1089/jicm.2022.0806, 36971836

[ref12] Kumari S. Dhapola R. Sharma P. Paidlewar M. Vellingiri B. Medhi B. . (2025). Unravelling neuronal death mechanisms: the role of cytokines and chemokines in immune imbalance in Alzheimer's disease progression. Ageing Res. Rev. 112:102883. doi: 10.1016/j.arr.2025.102883, 40885312

[ref13] Lim D. W. Park J. Han D. Lee J. Kim Y. T. Lee C. (2020). Anti-inflammatory effects of Asian fawn lily (Erythronium japonicum) extract on lipopolysaccharide-induced depressive-like behavior in mice. Nutrients 12:3809. doi: 10.3390/nu12123809, 33322645 PMC7764803

[ref14] Liu S. Gao J. Zhu M. Liu K. Zhang H. L. (2020). Gut microbiota and Dysbiosis in Alzheimer's disease: implications for pathogenesis and treatment. Mol. Neurobiol. 57, 5026–5043. doi: 10.1007/s12035-020-02073-3, 32829453 PMC7541367

[ref16] Loh J. S. Mak W. Q. Tan L. K. S. Ng C. X. Chan H. H. Yeow S. H. . (2024). Microbiota-gut-brain axis and its therapeutic applications in neurodegenerative diseases. Signal Transduct. Target. Ther. 9:37. doi: 10.1038/s41392-024-01743-1, 38360862 PMC10869798

[ref17] Ma Y. Y. Li X. Yu J. T. Wang Y. J. (2024). Therapeutics for neurodegenerative diseases by targeting the gut microbiome: from bench to bedside. Transl Neurodegener 13:12. doi: 10.1186/s40035-024-00404-1, 38414054 PMC10898075

[ref18] Mao Q. Zhang H. Zhang Z. Lu Y. Pan J. Guo D. . (2024). Co-decoction of Lilii bulbus and Radix Rehmannia Recens and its key bioactive ingredient verbascoside inhibit neuroinflammation and intestinal permeability associated with chronic stress-induced depression via the gut microbiota-brain axis. Phytomedicine 129:155510. doi: 10.1016/j.phymed.2024.155510, 38696921

[ref19] Marizzoni M. Mirabelli P. Mombelli E. Coppola L. Festari C. Lopizzo N. . (2023). A peripheral signature of Alzheimer's disease featuring microbiota-gut-brain axis markers. Alzheimer's Res. Ther. 15:101. doi: 10.1186/s13195-023-01218-5, 37254223 PMC10230724

[ref20] Mayer E. A. Nance K. Chen S. (2022). The gut-brain axis. Annu. Rev. Med. 73, 439–453. doi: 10.1146/annurev-med-042320-014032, 34669431

[ref21] Megur A. Baltriukiene D. Bukelskiene V. Burokas A. (2020). The microbiota-gut-brain Axis and Alzheimer's disease: Neuroinflammation is to blame? Nutrients 13:37. doi: 10.3390/nu13010037, 33374235 PMC7824474

[ref22] Mou Y. Du Y. Zhou L. Yue J. Hu X. Liu Y. . (2022). Gut microbiota interact with the brain through systemic chronic inflammation: implications on neuroinflammation, neurodegeneration, and aging. Front. Immunol. 13:796288. doi: 10.3389/fimmu.2022.796288, 35464431 PMC9021448

[ref23] Ohara T. E. Hsiao E. Y. (2025). Microbiota-neuroepithelial signalling across the gut-brain axis. Nat. Rev. Microbiol. 23, 371–384. doi: 10.1038/s41579-024-01136-9, 39743581

[ref24] Pan Q. Li Y. Q. Guo K. Xue M. Gan Y. Wang K. . (2021). Elderly patients with mild cognitive impairment exhibit altered gut microbiota profiles. J. Immunol. Res. 2021, 1–7. doi: 10.1155/2021/5578958, 34869782 PMC8635943

[ref25] Park H. R. Lee H. Cho W.-K. Ma J. Y. (2023). Pro-neurogenic effects of Lilii Bulbus on hippocampal neurogenesis and memory. Biomed. Pharmacother. 164:114951. doi: 10.1016/j.biopha.2023.114951, 37267636

[ref26] Qian X. Hai W. Chen S. Zhang M. Jiang X. Tang H. (2023). Multi-omics data reveals aberrant gut microbiota-host glycerophospholipid metabolism in association with neuroinflammation in APP/PS1 mice. Gut Microbes 15:2282790. doi: 10.1080/19490976.2023.2282790, 37992400 PMC10730179

[ref27] Qiu J. Zhang Y. Chen K. Xu J. Chen Y. Li M. . (2025). Integrating serum pharmacochemistry, network pharmacology, metabolomics and 16S rRNA sequencing to explore the mechanism of total flavonoids from Flemingia philippinensis in treating collagen induced arthritis rats. Phytomedicine 139:156531. doi: 10.1016/j.phymed.2025.156531, 39987603

[ref28] Ran Z. Ju B. Cao L. Hou Q. Wen L. Geng R. . (2023). Microbiome-metabolomics analysis reveals the potential effect of verbascoside in alleviating cognitive impairment in db/db mice. Food Funct. 14, 3488–3508. doi: 10.1039/d2fo03110h, 37000613

[ref29] Shi P. Zheng B. Cao Y. Niu G. Guo Q. (2025). Study on the mechanism of Trichosanthes kirilowii maxim. Against COPD based on serum chemical composition analysis, network pharmacology, and experimental study. Phytomedicine 140:156533. doi: 10.1016/j.phymed.2025.156533, 40023967

[ref30] Su Y. Liu N. Sun R. Ma J. Li Z. Wang P. . (2023). Radix Rehmanniae Praeparata (Shu Dihuang) exerts neuroprotective effects on ICV-STZ-induced Alzheimer's disease mice through modulation of INSR/IRS-1/AKT/GSK-3beta signaling pathway and intestinal microbiota. Front. Pharmacol. 14:1115387. doi: 10.3389/fphar.2023.1115387, 36843923 PMC9945319

[ref31] Sun Z. Z. Li X. Y. Wang S. Shen L. Ji H. F. (2020). Bidirectional interactions between curcumin and gut microbiota in transgenic mice with Alzheimer's disease. Appl. Microbiol. Biotechnol. 104, 3507–3515. doi: 10.1007/s00253-020-10461-x, 32095862

[ref32] Tang L. Liu J. Yang H. Zhao H. Q. Hu C. Ma S. J. . (2024). Microbiome Metabolomic analysis of the anxiolytic effect of Baihe Dihuang decoction in a rat model of chronic restraint stress. Drug Des. Devel. Ther. 18, 2227–2248. doi: 10.2147/DDDT.S458983, 38882046 PMC11180446

[ref33] Tao Q. Liang Q. Fu Y. Qian J. Xu J. Zhu Y. . (2024). Puerarin ameliorates colitis by direct suppression of macrophage M1 polarization in DSS mice. Phytomedicine 135:156048. doi: 10.1016/j.phymed.2024.156048, 39326132

[ref34] Varesi A. Pierella E. Romeo M. Piccini G. B. Alfano C. Bjorklund G. . (2022). The potential role of gut microbiota in Alzheimer's disease: from diagnosis to treatment. Nutrients 14:668. doi: 10.3390/nu14030668, 35277027 PMC8840394

[ref35] Wang S. Ma Y. Huang Y. Hu Y. Huang Y. Wu Y. (2022). Potential bioactive compounds and mechanisms of Fibraurea recisa Pierre for the treatment of Alzheimer's disease analyzed by network pharmacology and molecular docking prediction. Front. Aging Neurosci. 14:1052249. doi: 10.3389/fnagi.2022.1052249, 36570530 PMC9772884

[ref36] Wang H. Yang F. Gao Z. Cheng Z. Liang X. (2025). The gut-brain axis in Alzheimer's disease: how gut microbiota modulate microglial function. Front Aging 6:1704047. doi: 10.3389/fragi.2025.1704047, 41356558 PMC12678305

[ref37] Wei H. Yu C. Zhang C. Ren Y. Guo L. Wang T. . (2023). Butyrate ameliorates chronic alcoholic central nervous damage by suppressing microglia-mediated neuroinflammation and modulating the microbiome-gut-brain axis. Biomed. Pharmacother. 160:114308. doi: 10.1016/j.biopha.2023.114308, 36709599

[ref38] Xiao H. H. Chen J. C. Li H. Li R. H. Wang H. B. Song H. P. . (2022). Icarisid II rescues cognitive dysfunction via activation of Wnt/beta-catenin signaling pathway promoting hippocampal neurogenesis in APP/PS1 transgenic mice. Phytother. Res. 36, 2095–2108. doi: 10.1002/ptr.7430 35230733

[ref39] Yang X. Yu D. Xue L. Li H. Du J. (2020). Probiotics modulate the microbiota-gut-brain axis and improve memory deficits in aged SAMP8 mice. Acta Pharm. Sin. B 10, 475–487. doi: 10.1016/j.apsb.2019.07.001, 32140393 PMC7049608

[ref40] Zhang T. Gao G. Kwok L. Y. Sun Z. (2023). Gut microbiome-targeted therapies for Alzheimer's disease. Gut Microbes 15:2271613. doi: 10.1080/19490976.2023.2271613, 37934614 PMC10631445

[ref42] Zhu H. Shen F. Wang X. Qian H. Liu Y. (2024). Chlorogenic acid improves the cognitive deficits of sleep-deprived mice via regulation of immunity function and intestinal flora. Phytomedicine 123:155194. doi: 10.1016/j.phymed.2023.155194, 37995532

[ref43] Zou B. Li J. Ma R. X. Cheng X. Y. Ma R. Y. Zhou T. Y. . (2023). Gut microbiota is an impact factor based on the brain-gut axis to Alzheimer's disease: a systematic review. Aging Dis. 14, 964–1678. doi: 10.14336/AD.2022.1127, 37191418 PMC10187701

